# Establishing macroecological trait datasets: digitalization, extrapolation, and validation of diet preferences in terrestrial mammals worldwide

**DOI:** 10.1002/ece3.1136

**Published:** 2014-06-16

**Authors:** Wilm Daniel Kissling, Lars Dalby, Camilla Fløjgaard, Jonathan Lenoir, Brody Sandel, Christopher Sandom, Kristian Trøjelsgaard, Jens-Christian Svenning

**Affiliations:** 1 Institute for Biodiversity and Ecosystem Dynamics (IBED), University of AmsterdamP.O. Box 94248, 1090 GE, Amsterdam, The Netherlands; 2 Section for Wildlife Ecology and Section for Biodiversity, Department of Bioscience, Aarhus UniversityGrenåvej 14, DK-8410, Rønde, Denmark; 3 Unité de Recherche Ecologie et Dynamique des Systèmes Anthropisés (EDYSAN, FRE 3498 CNRS-UPJV), Université de Picardie Jules Verne1 Rue des Louvels, F-80037, Amiens Cedex, France; 4 Section for Ecoinformatics & Biodiversity, Department of Bioscience, Aarhus UniversityNy Munkegade 114, DK-08000, Aarhus C, Denmark; 5 Department of Zoology, University of Oxford, Wildlife Conservation Research Unit, The Recanati-Kaplan CentreTubney House, Abingdon Road, Tubney, Abingdon, OX13 5QL, U.K; 6 Section for Genetics, Ecology and Evolution, Department of Bioscience, Aarhus UniversityNy Munkegade 114, DK-8000, Aarhus C, Denmark

**Keywords:** Diet ecology, ecological trait data, feeding guild, Mammalia, phylogenetic conservatism, trophic structure

## Abstract

Ecological trait data are essential for understanding the broad-scale distribution of biodiversity and its response to global change. For animals, diet represents a fundamental aspect of species’ evolutionary adaptations, ecological and functional roles, and trophic interactions. However, the importance of diet for macroevolutionary and macroecological dynamics remains little explored, partly because of the lack of comprehensive trait datasets. We compiled and evaluated a comprehensive global dataset of diet preferences of mammals (“MammalDIET”). Diet information was digitized from two global and cladewide data sources and errors of data entry by multiple data recorders were assessed. We then developed a hierarchical extrapolation procedure to fill-in diet information for species with missing information. Missing data were extrapolated with information from other taxonomic levels (genus, other species within the same genus, or family) and this extrapolation was subsequently validated both internally (with a jack-knife approach applied to the compiled species-level diet data) and externally (using independent species-level diet information from a comprehensive continentwide data source). Finally, we grouped mammal species into trophic levels and dietary guilds, and their species richness as well as their proportion of total richness were mapped at a global scale for those diet categories with good validation results. The success rate of correctly digitizing data was 94%, indicating that the consistency in data entry among multiple recorders was high. Data sources provided species-level diet information for a total of 2033 species (38% of all 5364 terrestrial mammal species, based on the IUCN taxonomy). For the remaining 3331 species, diet information was mostly extrapolated from genus-level diet information (48% of all terrestrial mammal species), and only rarely from other species within the same genus (6%) or from family level (8%). Internal and external validation showed that: (1) extrapolations were most reliable for primary food items; (2) several diet categories (“Animal”, “Mammal”, “Invertebrate”, “Plant”, “Seed”, “Fruit”, and “Leaf”) had high proportions of correctly predicted diet ranks; and (3) the potential of correctly extrapolating specific diet categories varied both within and among clades. Global maps of species richness and proportion showed congruence among trophic levels, but also substantial discrepancies between dietary guilds. MammalDIET provides a comprehensive, unique and freely available dataset on diet preferences for all terrestrial mammals worldwide. It enables broad-scale analyses for specific trophic levels and dietary guilds, and a first assessment of trait conservatism in mammalian diet preferences at a global scale. The digitalization, extrapolation and validation procedures could be transferable to other trait data and taxa.

## Introduction

With the emergence of the macroecological research field (Brown and Maurer 1989), an increasing interest has developed in compiling comprehensive data on the geographic distribution of life on Earth. For instance, broad-scale datasets on species distributions, phylogenies, and ecological or life-history traits are now increasingly becoming electronically available, at least for some vertebrate groups such as birds and mammals (Bininda-Emonds et al. 2008; Jones et al. 2009; BirdLife International & NatureServe 2011; Jetz et al. 2012; IUCN 2013). However, compiling ecological trait data for species-rich clades is challenging and time-consuming, and many individual researchers lack the resources and time to compile such comprehensive datasets. Moreover, ecological trait data are often incomplete, even for well-known and well-studied clades (Jones et al. 2009) or for species in well-surveyed regions (Tyler et al. 2012). However, these trait data are essential for better understanding macroecological patterns (MacArthur 1972; Kissling et al. 2012; Barnagaud et al. 2014), evolutionary history (Cantalapiedra et al. 2014; Morlon 2014), or biodiversity and ecosystem functioning (McGill et al. 2006; Safi et al. 2011). Hence, new approaches are needed to get a better coverage of missing trait data, e.g. by “filling in” missing data with predicted values based on species for which trait data are available (Shan et al. 2012).

Diet represents a fundamental aspect of a species’ ecological niche (Simberloff and Dayan 1991). It constrains metabolic rates of organisms (Brown et al. 2004) and defines the functional roles and trophic interactions of species in ecosystems (Duffy 2002). Diet preferences can be important for understanding diversification (Price et al. 2012; Cantalapiedra et al. 2014), macroecological distributions (Kissling et al. 2009, 2012), as well as character displacement and evolutionary divergence of species (Grant and Grant 2006; Meiri et al. 2007). More generally, diet preferences have played an important role in understanding the ecology and evolution of communities (Hutchinson 1959; Cody and Diamond 1975; Burness et al. 2001). Nevertheless, only a few studies have examined latitudinal, environmental and biogeographic variation of diet preferences at a global scale (Hillebrand 2004; Primack and Corlett 2005; Kissling et al. 2009, 2012; Sandom et al. 2013; Barnagaud et al. 2014). Moreover, macroevolutionary studies have rarely integrated diet preferences or other trait data across species-rich clades (Morlon 2014). Hence, the importance of diet for macroevolutionary and macroecological dynamics and the structure and functioning of ecosystems worldwide remains little explored.

**Figure 1:**
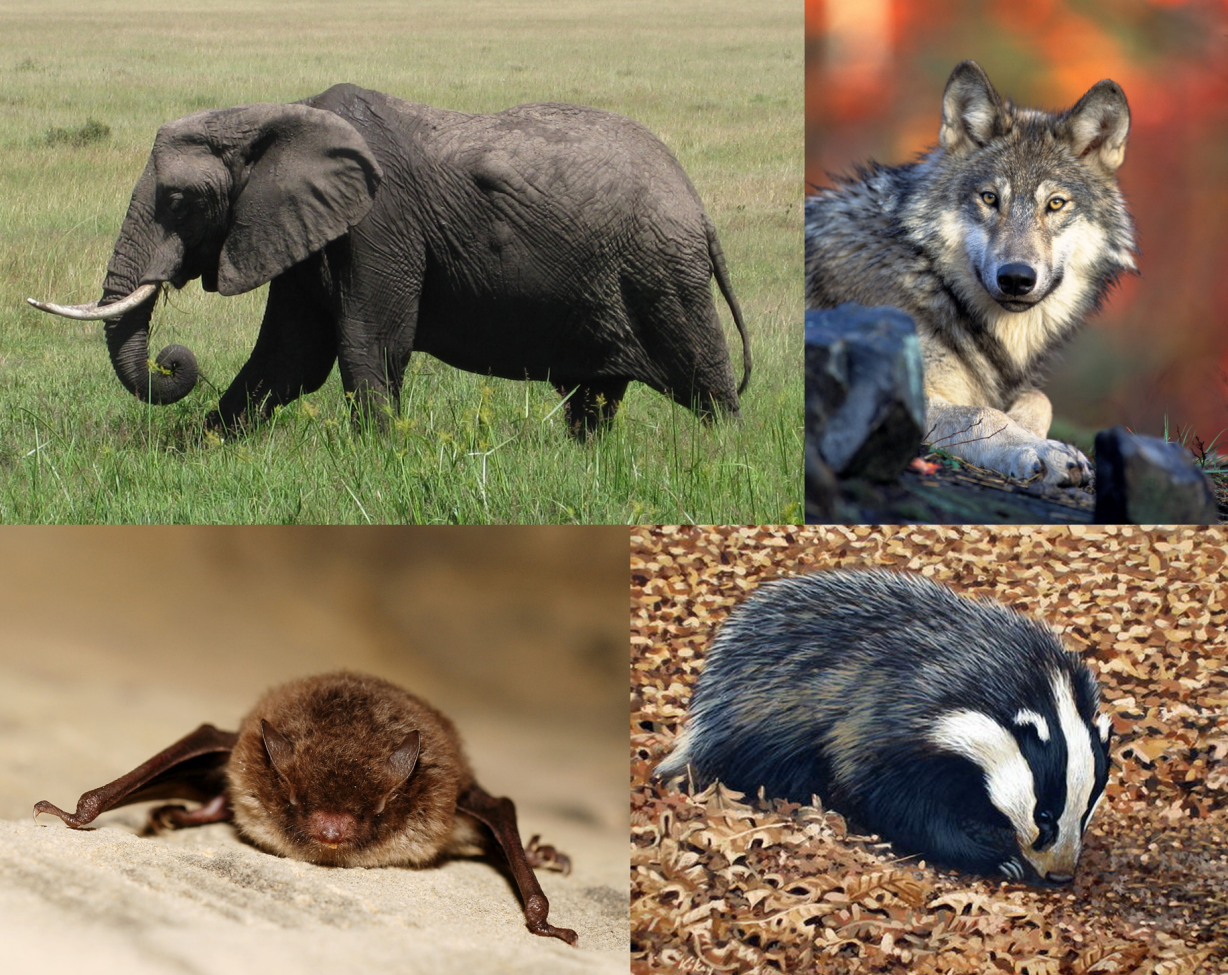
Four terrestrial mammal species representing different diet preferences. Upper left: African Elephant (*Loxodonta africana*), a typical herbivore. Upper right: the Gray Wolf (*Canis lupus*), a carnivore. Lower left: the Daubenton's Bat (*Myotis daubentonii*), an insectivore. Lower right: the European Badger (*Meles meles*), an omnivore. Photo credits: W. Daniel Kissling (elephant), Gary Kramer (wolf), Gilles San Martin (bat), Kókay Szabolcs (badger). The latter three were obtained from Wikimedia Commons (http://commons.wikimedia.org).

Mammals are a diverse vertebrate group whose species have colonized nearly all parts of the world. Mammalian species show a wide range of diet preferences (Fig. 1) which is partly related to their dental diversity (Price et al. 2012). Data on global species distributions (IUCN 2013) and phylogenetic relationships (e.g., Bininda-Emonds et al. 2008; Fritz et al. 2009) of mammals have recently become available and numerous ecological adaptations and life-history traits have been described in the literature (e.g., Nowak 1999; Smith et al. 2003; Jones et al. 2009; Qian et al. 2009; IUCN 2013). However, current datasets on ecological traits of mammals are incomplete and do not provide data for all mammals worldwide (e.g., Smith et al. 2003; Jones et al. 2009; Safi et al. 2011; Price et al. 2012). Available datasets on diet preferences of mammals are either restricted to small subsets of species (e.g., Cantalapiedra et al. 2014) or cover around 30–40% of the species (e.g., Jones et al. 2009; Price et al. 2012) and typically only allow categorizing species into three predefined trophic levels (carnivores, omnivores, herbivores). This forces researchers to limit their investigation to the best-known subset of taxa and to a few broad diet adaptations. Moreover, the deletion of missing values (or the use of incomplete datasets) reduces the power of statistical inference and might increase estimation bias (Nakagawa and Freckleton 2008). Hence, available data on key mammalian traits such as diet require additional efforts to achieve broader taxonomic coverage and finer ecological detail.

**Figure 2:**
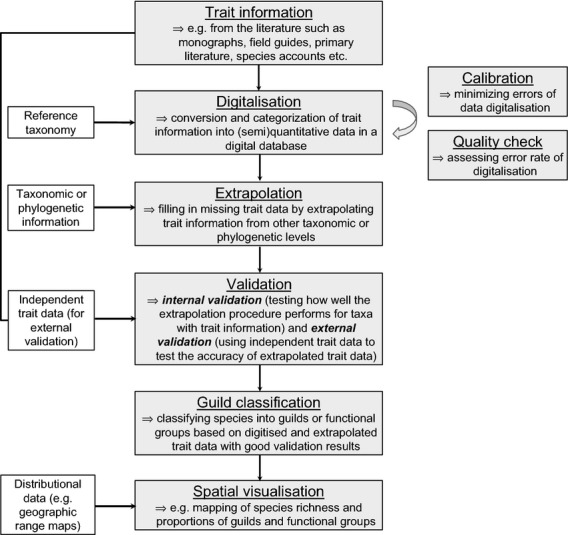
Schematic overview of how macroecological trait datasets can be established. Trait information from the literature is converted into a digital database (digitalization) and errors can be minimized and assessed during the digitalization process (calibration and quality check). Missing trait data are then filled from other taxonomic or phylogenetic levels (extrapolation). The performance of the extrapolation procedure can afterward be assessed internally and externally (validation). Finally, guilds or functional groups are classified (guild classification) and spatially mapped (spatial visualization). Grey boxes (middle and right) illustrate the key processes in the establishment of macroecological trait datasets, whereas white boxes (left) illustrate datasets that are additionally needed.

Here, we compiled and evaluated a global diet dataset for terrestrial mammals (referred to as “MammalDIET”, for general information see Table 1). We first digitized diet information from two comprehensive, global and cladewide data sources that provide a relatively standardized way of presenting mammalian diet information (Nowak 1999; IUCN 2013). We then quantified the consistency of data entry by multiple data recorders and developed an extrapolation procedure to fill-in missing diet information at the species level. Extrapolation was performed by using available diet knowledge from other species or other taxonomic levels (genus, family). We then validated the extrapolation procedure (both internally and externally) to identify the most reliable diet categories for classifying mammal species into trophic levels and dietary guilds. Finally, the frequency of different trophic levels and dietary guilds within mammalian families and orders was quantified and their species richness and proportion were mapped at a global scale. With the developed methodological framework (summarized as a flowchart in Fig. 2), we estimated diet preferences for nearly all terrestrial mammal species worldwide. We further provide MammalDIET as a freely available resource to enable macroecological and macroevolutionary analyses, and we encourage researchers to use, test, apply, and refine this dataset in the future.

**Table 1:** General information and key attributes of MammalDIET

Key attribute	General information
Dataset content	Diet preferences of terrestrial mammals worldwide
Dataset name	MammalDIET_v1.0
Original data sources	Nowak (1999), IUCN (2013)
Period of study	Data were extracted in 2011–2012 from Nowak (1999) and in 2013 from IUCN (2013). Data extrapolation and validation was done in 2011–2013.
Latest update	May 2014 for the final format of all files.
Database size	5364 species.
Format and storage mode	ASCII text, tab delimited, not compressed.
Header information	Details of headers information is provided in Appendix Table S1.
Alphanumeric attributes	Mixed. See storage type in Appendix Table S1.
Special characters	If no information is available, this is indicated by “NA”.
Taxonomy	Follows the IUCN taxonomy (IUCN 2013). The IUCN taxonomy differs from Nowak (1999) and synonyms were used to account for recent splits and lumps, and for cases where the spelling of names differed between sources. See taxonomic notes in Appendix Table S1.
Availability	MammalDIET is available from the Dryad Digital Repository: http://doi.org/10.5061/dryad.6cd0v

## Materials and Methods

### Digitalization of data

To compile diet information of mammals (“trait information” in Fig. 2), we used two key data sources on diet preferences of mammals worldwide (Nowak 1999; IUCN 2013). We focused on these two data sources because they contain global and cladewide knowledge on mammalian diets and because they allow a reasonably homogenous and standardized way of recording summary knowledge of mammalian diets. Diet information was first digitized from Nowak (1999) during 2011–2012 and additional information was added from IUCN (2013) during 2013 for species which had no species-level data from Nowak (1999). In all cases, we used the IUCN taxonomy as a reference taxonomy (IUCN 2013) and searched for synonyms where names differed between sources. We excluded marine families, but included all terrestrial mammal species (*n* = 5364). We converted written text descriptions (for examples see Table 2) of diet preferences from the two literature sources into ordinal data (ranks 1–3). In cases where the text did not allow inferring the relative importance of diet categories, we entered rank 1 assuming that these food items were equally important. In a few cases, the IUCN (2013) data source described species as, for example, carnivorous, herbivorous, frugivorous, omnivorous for which we recorded rank 1 in the corresponding diet categories. A zero (rank 0) was assigned if a specific diet category was not recorded in the literature for a given taxon. Moreover, we took a conservative approach and recorded diet information only at the specific taxonomic level of the original data source, that is, at species, genus, or family level. The majority of diet information from Nowak (1999) was available at the genus level, and hence, we digitized this information at the genus level, not at the species level, even if species within a genus are likely to have the same diets. In contrast, the IUCN (2013) data were almost exclusively available as species-level information.

**Table 2:** Examples of text descriptions and keywords on the relative importance of mammalian diet categories as reported in two literature sources with global, cladewide coverage (Nowak 1999; IUCN 2013). For data entry into MammalDIET, text descriptions were converted into ordinal data (rank 1–3)

Diet rank	Ecological interpretation	Examples of text descriptions
Rank 1	Primary food items representing diet categories with major importance (the main food items consumed)	Consists mainly, feeds mostly, concentrates, includes, major portion, prefers, especially significant, most frequently consumed, almost exclusively
Rank 2	Secondary food items representing diet categories of intermediate importance	But also includes, may include, also important, feeds partly, also feeds
Rank 3	Occasional food items representing diet categories of little importance (i.e., rarely eaten)	Occasionally, sometimes, small amounts, supplemented by, a few, rarely, opportunistically

**Figure 3:**
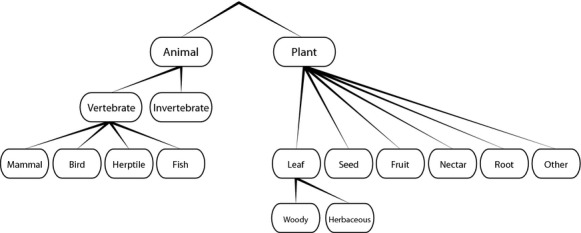
Hierarchical structure of the sixteen diet categories used for data entry into MammalDIET. Diet categories represent four different hierarchical levels of diet information. Detailed information about these diet categories is provided in Appendix Table S1.

For digitizing the data (“digitalization” in Fig. 2), a total of sixteen diet categories at four hierarchical levels were distinguished (Fig. 3). At the first and coarsest level, we distinguished between “Animal” and “Plant.” At the second level, the animal category was subdivided into “Vertebrate” and “Invertebrate.” At the third level, the plant category was subdivided into “Seed”, “Fruit”, “Nectar”, “Root”, “Leaf”, and “Other” material, and the vertebrate category was subdivided into “Mammal”, “Bird”, “Herptile” (amphibians and reptiles), and “Fish.” At the fourth and finest level, we subdivided the leaf category into leaves from woody plants (“Woody”) and leaves from herbaceous plants (“Herbaceous”). This was carried out to allow the division of mammalian herbivores into browsers and grazers. For all data entry, we recorded diet preferences down to the finest diet categories possible.

At the beginning of the data digitalization process, we tested how well diet descriptions from the data sources could be converted into diet ranks in MammalDIET. To assure the consistency of data entry by multiple data recorders (all authors except J.-C.S.), we randomly selected 20 mammal species from Nowak (1999) before compiling the data and each data recorder then ranked diet descriptions from the source for the same sample species (“calibration” in right-hand side of Fig. 2). Discrepancies in data entries between data recorders were subsequently discussed among all persons to minimize errors of the digitalization process, that is, when transferring written diet descriptions from the sources into an ordinal scale in MammalDIET. Such a calibration step was used to standardize the digitizing of data by multiple recorders. After the data from Nowak (1999) had been assembled, an additional test (“quality check” in right-hand side of Fig. 2) was performed based on 120 randomly selected species out of those species for which diet data from Nowak (1999) were available at the species level (*n* = 682). The recorders re-entered data by transferring written descriptions from Nowak (1999) to diet ranks in MammalDIET and then calculated the percentage of correctly classified diet ranks for all diet categories across the 120 species. This allowed assessing the error rate due to data entry via multiple data recorders.

### Extrapolation of diet ranks

Not all diet information was available at the species level. Some information, especially in Nowak (1999), was only available at the genus or, more rarely, family level. We therefore developed a procedure to extrapolate diet information from other species or higher taxonomic levels (genus, family) to species without diet information (“extrapolation” in Fig. 2). This extrapolation procedure assumed some degree of phylogenetic conservatism in diet preferences, at least for the recorded diet categories and at the taxonomic levels applied. The different approaches to data extrapolation are explained in detail later. Information on how diet data were extrapolated is also provided for each species in Appendix Table S1 (cf. variable “FillCode”).

No data extrapolation was necessary for those species that already had species-level information from the two data sources (FillCode = 0). For the other species, data extrapolation was performed hierarchically. First, diet data were filled from the genus level (FillCode = 1), then from other species within the same genus (FillCode = 2.1 or 2.2) and finally from the family level (FillCode = 3). We distinguished two ways of data filling from other species within the same genus. First, if only one species in the genus had data, we applied this information to our missing species (FillCode = 2.1). Second, if more than one species in the genus had data, we assigned the diet information for each category to the missing species if all species had the same information in that category (FillCode = 2.2), that is, we only extrapolated information that was consistent among congeneric species. Otherwise data were assigned as not available (“NA”). This ensured a rather conservative way of extrapolating diet information to the species level.

### Internal validation of extrapolation

To investigate the robustness of the extrapolation procedure, we applied two validation procedures (“validation” in Fig. 2): first an internal validation (using the species-level data from the compiled dataset) and second an external validation (using an independent data source, see below). For the internal validation, we evaluated how well each of the species with species-level diet information in the compiled dataset (i.e., FillCode = 0; *n* = 2033 species) would be filled if no diet data were available. We used a jack-knife approach where diet ranks were removed from one focal species at a time and then filled by the same extrapolation procedure as described above. This predicted the diet ranks for the focal species as if there was no diet information available for that species. The predicted diet ranks of the focal species were then compared with the empirical diet data as recorded from the original data sources. Across all species in the validation subset, we then calculated the proportion of correctly predicted diet ranks (including ranks 1–3 and 0). Species that were the only species with diet information in a genus had to be disregarded for this internal validation if no further diet information was available at the genus or family level.

### External validation of extrapolation

We used an external validation to test the accuracy of the extrapolated species-level diet data in MammalDIET relative to diet data extracted from an additional, independent data source (see validation in Fig. 2). For this additional data source (“external validation dataset”), we chose the species accounts from the new edition of Mammals of Africa (Butynski et al. 2013; Happold 2013; Happold and Happold 2013; Kingdon and Hoffmann 2013a,b; Kingdon et al. 2013), a series of six volumes describing in detail every currently recognized species of terrestrial mammal in Africa. Although having a regional focus (African continent), this compilation of books is the most comprehensive, up-to-date species-level data source that is currently available for mammals in a specific biogeographic region. From the full list of all species with extrapolated diet information in our dataset (*n* = 3329), we first selected those occurring in Africa (*n* = 611) and then randomly selected species from this list to subsequently enter diet information from the Mammals of Africa. To aim for a reasonable sample size of species across different diet categories, we stratified the random selection by choosing 30 random species (if available) for each of the sixteen diet categories. Several species were selected more than once and we removed these duplicates and thus ended up with a total of 289 randomly selected species. For each of these species, we checked the diet information in Mammals of Africa and entered species-level diet information in the same way as for MammalDIET (if available). For each of the 16 diet categories (cf. Fig. 3), we then compared how often the diet information from the external validation dataset (Mammals of Africa) was consistent with the extrapolated species-level knowledge in MammalDIET. We report the percentage of correctly extrapolated diet ranks (separately for rank 1 only, and for rank 1 and 2 combined) for the 16 diet categories. We performed this validation for all species in the external validation dataset as well as separately for mammal orders with ≥15 species.

### Classification of trophic levels and dietary guilds

We used the information in MammalDIET to group mammal species into different trophic levels and dietary guilds. Based on the internal and external validation results, we identified various diet categories that were reliable for such a classification (for details see Results). We applied two different types of classifications. First, we used the “Animal” and “Plant” categories to classify species into three trophic levels (carnivores, herbivores, and omnivores; see “TrophicLevels” in Appendix S1). This classification was coarse and mutually exclusive so that species in one trophic level could not be present in the other trophic level. Second, a few of the diet categories (“Mammal”, “Invertebrate”, “Seed”, “Fruit”, and “Leaf”) were robust enough, given the validation procedures, to provide a finer classification into dietary guilds (mammal eaters, insectivores, granivores, frugivores, and folivores). This fine classification focused on the functional role of the species in the ecosystem, and categories were not mutually exclusive. After classification we examined how well trophic levels and dietary guilds were represented among mammal orders and families.

### Spatial visualization

To illustrate potential applications of the presented data, we combined the trophic level and dietary guild classification with data on the global distribution of mammals. We used the global species distribution maps for terrestrial mammal species from IUCN (2013). We converted the polygon range maps to rasters on a Behrmann cylindrical equal-area projection and extracted species occurrences for grid cells at a resolution of 2° equivalents (∼220 km). We chose 2° equivalents over 1° equivalents, but we note that statistical analyses with range maps at these two spatial resolutions usually give similar results (e.g., Hurlbert and Jetz 2007; Kissling et al. 2012). The data handling and extraction were similar to the procedure described by Sandom et al. (2013). We mapped the global distribution of species richness and proportions for each trophic level and dietary guild, excluding Antarctica and grid cells with <50% land area. We note that this mapping is only used for illustrative purposes and that more rigorous statistical analyses on potential drivers of these large-scale richness patterns need further scrutiny.

## Results

### Digitalization of data

Of the 5364 terrestrial mammal species, a total of 2033 species (38% of all terrestrial mammal species) had species-level information available from the two global data sources. Of those, 682 species were entered from Nowak (1999) and an additional 1351 species from IUCN (2013). Furthermore, information on diet preferences were also available for many genera (*n* = 453) and families (*n* = 32). These genus and family diet data were only used for the extrapolation procedure.

The calibration step before the data digitalization confirmed that minimizing discrepancies between multiple data recorders can be important when transferring written text descriptions into semi-quantitative ranks. The subsequent additional quality check of 120 randomly selected species revealed a relatively low error rate due to data entry via multiple data recorders. In most cases, diet ranks were identically re-entered for a specific diet category (overall mean ± SD across all categories: 94.41% ± 0.04%, *n* = 120). The least success was obtained for the diet category “Other” plant material (86%), whereas the highest success was obtained for the diet category “Root” and “Herbaceous” (>99%). All other diet categories had a high classification success of ≥90% (“Animal”: 94%; “Vertebrate”: 95%; “Mammal”: 96%; “Bird”: 96%; “Herptile”: 96%; “Fish”: 97%; “Invertebrate”: 91%; “Plant”: 90%; “Seed”: 96%; “Fruit”: 90%; “Nectar”: 96%; “Leaf”: 92%; “Woody”: 95%).

### Extrapolation of data

Among the 3331 species (62% of all terrestrial mammal species) with missing species-level diet data, a total of 2556 species (48%) were filled with diet information from the genus level (FillCode = 1). In addition, 337 species (6%) were filled from other species within the same genus (FillCode = 2.1 or 2.2). Of those, 266 species (5%) were filled with information available from one other species in the same genus (FillCode = 2.1), whereas 71 species (1%) were filled from more than one species in the genus (FillCode = 2.2). Finally, information from the family level was extrapolated to 436 species (8%, FillCode = 3). Hence, a total of 3329 species had extrapolated diet information, with only two species (*Echinoprocta rufescens* and *Prolagus sardus*) remaining without diet information after the extrapolation procedure. The former of these two species seems to be phylogenetically nested within the genus *Coendou* (Voss et al. 2013) and can therefore be considered as herbivorous, whereas the latter is extinct (IUCN 2013) and dental morphology suggests a predominantly herbaceous diet (Angelone 2005). Note that we did not enter this additional information into MammalDIET as it was not available from the two original data sources. In total, the original data together with the extrapolation procedure provided species-level data on diet preferences for 99.9% of the world's terrestrial mammals (*n* = 5362 species).

**Figure 4:**
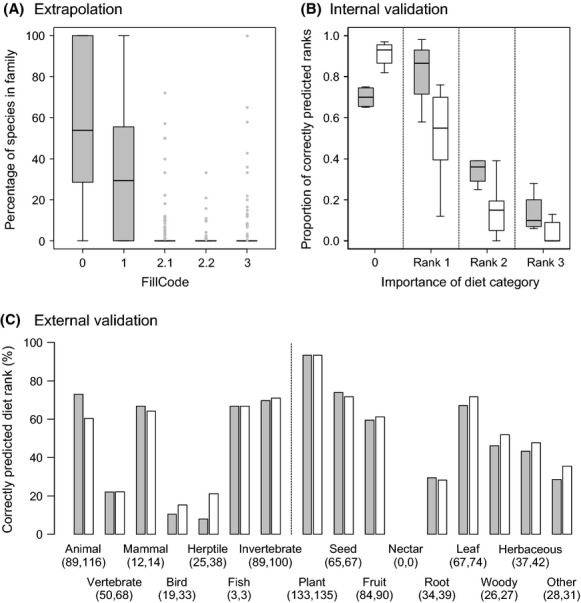
Summary of (A) extrapolation, (B) internal validation, and (C) external validation of global diet knowledge in mammals. In (A) each boxplot summarizes the percentage of species within mammal families (*n* = 140) according to how extrapolation of diet information was done (FillCode = 0, 1, 2.1, 2.2 and 3). Extrapolation was not necessary for those species (*n* = 2033) for which species-level information was already available from the data sources (FillCode = 0). For the other species (*n* = 3331), extrapolation was performed from the genus level (FillCode = 1), from one other species in the genus (FillCode = 2.1), from more than one species in the genus (FillCode = 2.2), or from family level (FillCode = 3). Raw data are provided in Appendix Table S2. In (B) each boxplot summarizes the proportion of correctly predicted diet ranks for high (grey boxes) and low (white boxes) hierarchical levels (compare Fig. 3). High hierarchical levels include the diet categories “Animal”, “Plant”, “Vertebrate”, and “Invertebrate”, whereas the low hierarchical levels include all other diet categories. Information on ranks 1–3 is provided in Table 2. The “0″ indicates that a diet category was not used (i.e., assumed absence). In (C), extrapolated diet data are validated independently with an external validation dataset (Mammals of Africa, see text for details). The percentage of correctly predicted diet ranks is given for each of the sixteen diet categories for rank 1 data only (gray bars) and for rank 1 and 2 data combined (white bars). Numbers below diet categories give the sample size (number of species) for each validation. Boxes in (A) and (B) represent the interquartile range (IQR), horizontal lines within the boxes represent medians, whiskers extend to 1.5 times the IQR, and outliers are plotted as dots.

Across all terrestrial mammal families, the percentage of species with different filling codes varied widely (Fig. 4A). However, species-level diet information was typically available for half of the species within a given family (median: 54%). Some families had species-level diet information for all species while a few families had zero coverage (range: 0–100%). Most diet information was extrapolated from the genus level (median: 29% of species across families). Across families, filling from just one other species in the genus (FillCode = 2.1), from all other species with diet information within the same genus (FillCode = 2.2), or from family level (FillCode = 3) was generally very low (Fig. 4). The extrapolation of diet information was also not homogenous across mammal orders (Table 3). Most diet information within orders was filled from genus level information (FillCode = 1). Species-level information (FillCode = 0) was particularly well represented in the mammal orders Carnivora (82%), Cetartiodactyla (66%), and Primates (59%) (Table 3).

**Table 3:** Summary information across mammal orders of how extrapolation of diet preferences was performed (FillCode = 0, 1, 2.1, 2.2, 3). Diet information was available for 2033 species at the species level from the original data sources (FillCode = 0). For the other species, diet data were first filled from the genus level (FillCode = 1, *n* = 2556 species), then from one other species (FillCode = 2.1, *n* = 266 species) or from more than one species within the same genus (FillCode = 2.2, *n* = 71 species), and finally from the family level (FillCode = 3, *n* = 436 species). See text for details

		Number of species (percentage) in FillCode				
		
Mammal order	Total number of species	0	1	2.1	2.2	3
Afrosoricida	54	15 (28)	36 (67)	3 (6)	0 (0)	0 (0)
Carnivora	249	203 (82)	35 (14)	4 (2)	7 (3)	0 (0)
Cetartiodactyla	244	160 (66)	65 (27)	13 (5)	3 (1)	3 (1)
Chiroptera	1150	449 (39)	555 (48)	21 (2)	26 (2)	99 (9)
Cingulata	21	4 (19)	17 (81)	0 (0)	0 (0)	0 (0)
Dasyuromorphia	74	20 (27)	52 (70)	0 (0)	0 (0)	2 (3)
Dermoptera	2	1 (50)	0 (0)	1 (50)	0 (0)	0 (0)
Didelphimorphia	95	39 (41)	50 (53)	6 (6)	0 (0)	0 (0)
Diprotodontia	146	41 (28)	99 (68)	6 (4)	0 (0)	0 (0)
Eulipotyphla	450	111 (25)	253 (56)	45 (10)	0 (0)	41 (9)
Hyracoidea	5	2 (40)	3 (60)	0 (0)	0 (0)	0 (0)
Lagomorpha	93	46 (49)	45 (48)	1 (1)	0 (0)	0 (0)
Macroscelidea	17	4 (24)	13 (76)	0 (0)	0 (0)	0 (0)
Microbiotheria	1	1 (100)	0 (0)	0 (0)	0 (0)	0 (0)
Monotremata	5	5 (100)	0 (0)	0 (0)	0 (0)	0 (0)
Notoryctemorphia	2	1 (50)	1 (50)	0 (0)	0 (0)	0 (0)
Paucituberculata	6	4 (67)	0 (0)	0 (0)	2 (33)	0 (0)
Peramelemorphia	22	6 (27)	11 (50)	2 (9)	3 (14)	0 (0)
Perissodactyla	16	13 (81)	3 (19)	0 (0)	0 (0)	0 (0)
Pholidota	8	7 (88)	0 (0)	0 (0)	0 (0)	1 (12)
Pilosa	10	4 (40)	5 (50)	0 (0)	0 (0)	1 (10)
Primates	415	243 (59)	150 (36)	21 (5)	1 (0)	0 (0)
Proboscidea	2	2 (100)	0 (0)	0 (0)	0 (0)	0 (0)
Rodentia	2256	647 (29)	1147 (51)	143 (6)	29 (1)	289 (13)
Scandentia	20	4 (20)	16 (80)	0 (0)	0 (0)	0 (0)
Tubulidentata	1	1 (100)	0 (0)	0 (0)	0 (0)	0 (0)
Total	5364	2033 (38)	2556 (47)	266 (5)	71 (1)	436 (8)

### Internal validation of extrapolation

The internal validation with the compiled dataset showed that the extrapolation procedure performed best for primary food items (rank 1, Fig. 4), especially for coarse diet categories such as “Animal” and “Plant” (Table 4). More generally, diet categories at high hierarchical levels (“Animal”, “Plant”, “Vertebrate”, and “Invertebrate”) were on average better predicted than those at low hierarchical levels (all other categories) (Fig. 4). However, a few diet categories at low hierarchical levels (“Mammal”, “Seed”, “Fruit”, and “Leaf”) also showed good validation results for rank 1 (Table 4), whereas the “Vertebrate” category had the lowest predictive potential among the higher hierarchical levels (Table 4). The prediction of the absence of a diet category (rank 0) was generally very good (Fig. 4) and tended to be better for low hierarchical levels than for high taxonomic levels (opposite to ranks 1–3; Fig. 4).

**Table 4:** Internal validation of extrapolating diet information, illustrated by the proportions of correctly predicted diet ranks (rank 0–3) within a subset of species for which species-level diet information was available (*n* = 2033 species). Prediction of diet ranks was performed using a jack-knife approach that first removed the original diet information of a focal species and then predicted the diet ranks with a filling procedure as described in the main text. Proportions >0.60 are highlighted in bold. “NA” reflects missing diet rank data in a specific diet category

	Proportions of correctly predicted diet ranks			
	
Diet category	0	1	2	3
Animal	**0.65**	**0.88**	0.39	0.08
Vertebrate	**0.75**	0.58	0.25	0.28
Mammal	**0.95**	**0.75**	0.05	0.13
Bird	**0.97**	0.38	0.05	0.24
Herptile	**0.96**	0.12	0.06	0
Fish	**0.93**	0.43	0	0.09
Invertebrate	**0.66**	**0.85**	0.39	0.06
Plant	**0.74**	**0.98**	0.33	0.12
Seed	**0.88**	**0.69**	0.19	0.03
Fruit	**0.82**	**0.76**	0.18	0
Nectar	**0.97**	0.43	0.38	NA
Root	**0.93**	**0.67**	0.14	0
Leaf	**0.85**	**0.71**	0.2	0
Woody	**0.93**	0.33	0.16	NA
Herbaceous	**0.93**	**0.68**	0	NA
Other	**0.83**	0.41	0.39	0

The ability to correctly predict primary food items (rank 1) varied from 12–98% (median: 68%) across the sixteen diet categories (Table 4). Predictions of secondary food items (rank 2) were much lower (median: 19%; range: 0–39%) and occasional food items (rank 3) were generally difficult to predict (Table 4, Fig. 4). Hence, the internal validation showed that (1) extrapolations were most reliable for primary food items (i.e., rank 1) and (2) that several diet categories (“Animal”, “Plant”, “Invertebrate”, “Mammal”, “Seed”, “Fruit”, and “Leaf”) had good potential for extrapolating diet information to the species level.

### External validation of extrapolation

Of 289 African species in the external validation dataset, 163 species (56%) had species-level diet information available from the six volumes of Mammals of Africa. The number of species for validating the sixteen different diet categories was generally good (>10 species) although two diet categories (“Fish”, “Nectar”) had insufficient sample sizes (3 and 0 species, respectively). For diet categories with sufficient sample sizes, the external validation showed that three diet categories at high hierarchical levels (“Animal”, “Plant”, and “Invertebrate”) as well as four diet categories at low hierarchical levels (“Mammal”, “Seed”, “Fruit”, “Leaf”) correctly predicted the diet ranks in ≥60% of the cases (Fig. 4C). The same diet categories were also identified with good validation scores by the internal validation.

**Figure 5:**
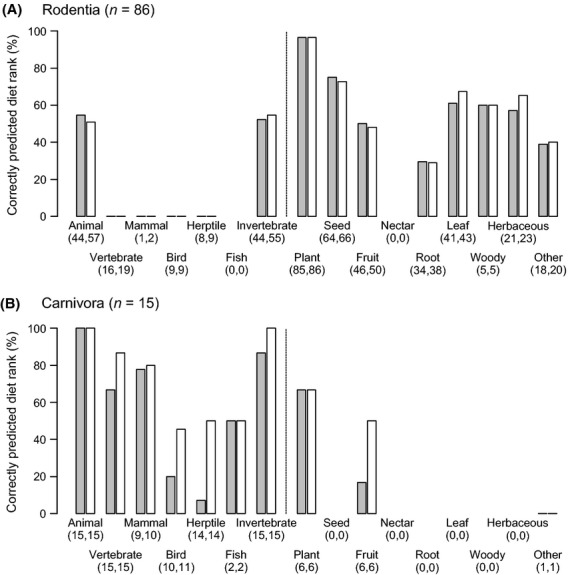
External validation of extrapolating diet knowledge for two mammal orders that contain species which use a broad range of either plant or animal diet categories. (A) Rodentia (here rodents such as African dormice, gerbils, mice, etc.) are predominantly herbivorous and insectivorous, but different species use different plant diet categories. (B) Carnivora (mostly represented here by genets and mongooses) predominantly feed on animal material, but the importance of different animal diet categories varies among species. Bars illustrate the percentage of correctly predicted diet ranks of each of the 16 diet categories for rank 1 data (gray bars) and for rank 1 and 2 data combined (white bars). Numbers below diet categories give the sample size (number of species) for each validation. The results of this external validation are based on the Mammals of Africa (see text for details).

**Figure 6:**
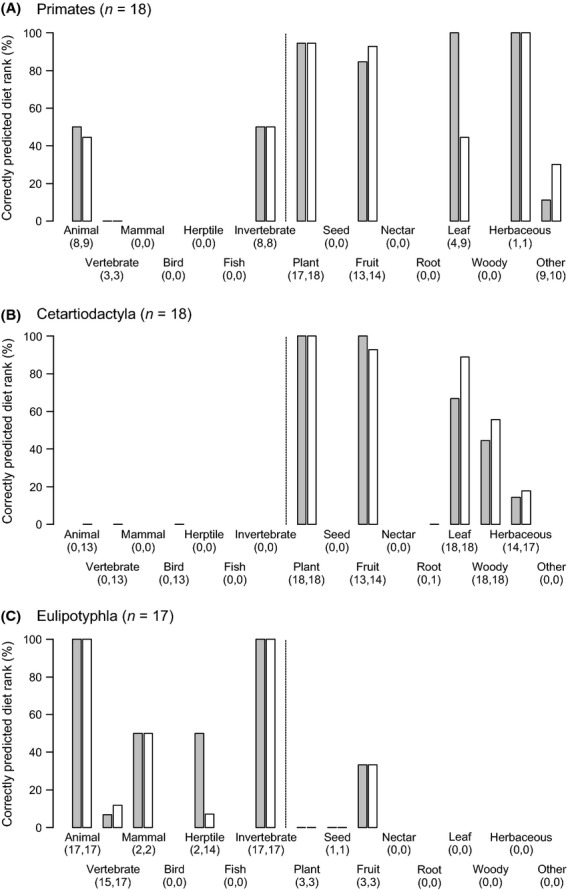
External validation of extrapolating diet knowledge for three mammal orders that contain species which feed on a few diet categories. (A) Primates (here mostly monkeys and galagos) feed on “Plant” and “Fruit”, but the use of leaves and invertebrates varies among species. (B) Cetartiodactyla (here duikers, dik-diks, etc.) are herbivores with a specialization on fruits and leaves, but being a browser (“Woody” leaves) or a grazer (“Herbaceous” leaves) varies among species. (C) Eulipotyphla (shrews) are highly insectivorous (incl. invertebrates) with other food items being only eaten by a few species. Bars illustrate the percentage of correctly predicted diet ranks of each of the 16 diet categories for rank 1 data (gray bars) and for rank 1 and 2 data combined (white bars). Numbers below diet categories give the sample size (number of species) for each validation. The results of this external validation are based on the Mammals of Africa (see text for details).

To explore taxonomic variation in extrapolating diet ranks, we examined the results from the external validation separately for each of five mammal orders with ≥15 species (Figs 5 and 6). This revealed interesting differences in the potential to predict diet adaptations both within and among clades. Two orders (Rodentia and Carnivora) showed a broad range of diet categories, but their specialization on plants and animals differed. Rodents (Rodentia), being predominantly herbivorous and insectivorous and representing the most species-rich order in the external validation dataset (as well as globally, Table 3), showed a 100% prediction accuracy for the diet category “Plant”, but a mixed picture with varying percentages of correctly predicted diet ranks for other categories (Fig. 5A). The order Carnivora (here mostly represented by genets and mongooses), predominantly feeding on animal material, showed very good predictions (usually >75% correctly predicted diet ranks) for the categories “Animal”, “Vertebrate”, “Mammal”, and “Invertebrate”, but lower values for other diet categories (Fig. 5B). In contrast to the broad range of diet categories in Rodentia and Carnivora, the three other mammal orders showed a stronger specialization on a few specific diet categories (Fig. 6). Primates showed excellent evaluation scores for “Plant” and “Fruit”, but lower scores for “Animal” and “Invertebrate” (Fig. 6A). The herbivorous Cetartiodactyla (here mostly duikers, dik-diks, etc.) also showed excellent evaluation scores for “Plant” and “Fruit” (Fig. 6B), but whether species were browsers or grazers varied among species (i.e., lower scores for “Woody” and “Herbaceous” leaves). Finally, the highly insectivorous Eulipotyphla (shrews) showed excellent predictions for “Animal” and “Invertebrate”, whereas other diet categories were only represented among a few species (Fig. 6C).

### Classification of trophic levels and dietary guilds

Based on the internal and external validation results above, two classification procedures were applied (for details see Table 5). First, each species was grouped into one of three trophic levels: carnivores, herbivores, and omnivores. These mutually exclusive trophic levels were based on the two coarsest diet categories (“Animal” and “Plant”) because they defined the highest hierarchical level (Fig. 3) and were among the diet categories with the best validation scores (rank 1 in Table 4 and Fig. 4C). Only 13 species (0.24%) could not be allocated (“Not assigned” in Table 5) according to this classification.

**Table 5:** Ecological and technical details of defining trophic levels and dietary guilds of mammals. Internal and external validations of correctly extrapolating diet ranks were used to guide which diet categories were reliable to group species into different trophic levels and dietary guilds (see text for details). The trophic levels represent three mutually exclusive groups (carnivores, herbivores, omnivores) based on diet categories at the highest hierarchical level (“Animal”, “Plant”). The five dietary guilds (mammal eaters, insectivores, granivores, frugivores, folivores) are not mutually exclusive and were classified based on fine diet categories (“Mammal”, “Invertebrate”, “Seed”, “Fruit”, “Leaf”) with good validation scores (compare Table 4)

Classification	Ecological definition	Technical definition	Number of species (%)
Trophic levels			
Carnivores	Species predominantly eating animals, but not or only occasionally eating plants	Rank 1 or 2 in “Animal” category, rank 0 or 3 in “Plant” category	1637 (31)
Herbivores	Species predominantly eating plant material, but not or only occasionally animals	Rank 1 or 2 in “Plant” category, rank 0 or 3 in “Animal” category	1926 (36)
Omnivores	Species feeding on both animals and plants	Rank 1 or 2 in “Animal” and “Plant” category, respectively	1788 (33)
Not assigned	Species which do not fit into other trophic levels	Remaining species	13 (0)
Dietary guilds			
Mammal eaters	Species which have mammals as an important part of their diet	Rank 1 in “Mammal” category, all other categories possible	209 (4)
Insectivores	Species which have insects or invertebrates as an important part of their diet	Rank 1 in “Invertebrate” category, all other categories possible	2821 (53)
Granivores	Species which have seeds as an important part of their diet	Rank 1 in “Seed” category, all other categories possible	1204 (22)
Frugivores	Species which have fruits as an important part of their diet	Rank 1 in “Fruit” category, all other categories possible	1692 (32)
Folivores	Species which have leaves as an important part of their diet	Rank 1 in “Leaf” category, all other categories possible	1473 (27)

In a second classification, we used finer diet categories (i.e., all categories below “Animal” and “Plant”, Fig. 3) to provide a more detailed classification for specific dietary guilds. For this second classification, we only used diet categories if they had well predicted diets in the internal validation (i.e., proportion predicted >0.60 for both rank 0 and rank 1, Table 4) as well as good validation scores in the external validation (≥60% correctly predicted diet ranks, compare Fig. 4C) for diet categories with sufficient sample sizes (>10 species). This included the diet categories “Mammal”, “Invertebrate”, “Seed”, “Fruit”, and “Leaf”. Hence, we classified — for each of these diet categories — species into dietary guilds (mammal eaters, insectivores, granivores, frugivores, and folivores) if the respective diet category had a rank 1 in a given species (Table 5). These dietary guilds were not mutually exclusive because a species could be classified into more than one dietary guild (e.g., granivore, frugivore) if it had a rank 1 in these diet categories (“Seed”, “Fruit”). A detailed overview of the two classifications is provided in Table 5. The dietary guild assignment for each species is also provided with the dataset (Appendix Table S1, dataset available from the Dryad Digital Repository: http://doi.org/10.5061/dryad.6cd0v).

The percentage of species within trophic levels and dietary guilds varied considerably among mammal orders (Table 6). For instance, the largest proportions of carnivorous species (as defined in Table 5) were found in the mammal orders Dasyuromorphia (a group of Australian marsupials), Eulipotyphla (such as shrews), and Afrosoricida (tenrecs, otter-shrews and golden-moles). For herbivores, the orders Cetartiodactyla (such as bovids and deer) and Lagomorpha (such as hares and rabbits) contained the highest proportions of species. Omnivorous species were best represented within the orders Didelphimorphia (opossums) and Scandentia (treeshrews). Dietary guilds included mammal eaters (e.g., felids and canids), insectivores (e.g., microbats, tenrecs, shrews), frugivores (e.g., some groups of bats and primates), granivores (e.g., some groups of rodents), and folivores (e.g., bovids, kangaroos, and hares). A detailed overview of trophic levels and dietary guilds is provided for mammal orders in Table 6 and for mammal families in Appendix Table S3.

**Table 6:** Distribution of species within trophic levels and dietary guilds for each mammal order. For definition see Table 5

		Number of species (percentages) within trophic levels and dietary guilds								
		
		Trophic levels				Dietary guilds				
			
Mammal order	Total species number	Carnivores	Herbivores	Omnivores	Not assigned	Mammal eaters	Insectivores	Granivores	Frugivores	Folivores
Afrosoricida	54	53 (98)	0 (0)	1 (2)	0 (0)	1 (2)	54 (100)	0 (0)	0 (0)	0 (0)
Carnivora	249	139 (56)	5 (2)	105 (42)	0 (0)	146 (59)	129 (52)	8 (3)	67 (27)	6 (2)
Cetartiodactyla	244	0 (0)	216 (89)	28 (11)	0 (0)	0 (0)	9 (4)	22 (9)	65 (27)	226 (93)
Chiroptera	1150	809 (70)	252 (22)	89 (8)	0 (0)	3 (0)	852 (74)	6 (1)	293 (25)	2 (0)
Cingulata	21	12 (57)	0 (0)	9 (43)	0 (0)	0 (0)	19 (90)	0 (0)	2 (10)	2 (10)
Dasyuromorphia	74	67 (91)	0 (0)	7 (9)	0 (0)	20 (27)	62 (84)	0 (0)	0 (0)	0 (0)
Dermoptera	2	0 (0)	2 (100)	0 (0)	0 (0)	0 (0)	0 (0)	0 (0)	0 (0)	2 (100)
Didelphimorphia	95	11 (12)	2 (2)	82 (86)	0 (0)	26 (27)	91 (96)	19 (20)	65 (68)	1 (1)
Diprotodontia	146	2 (1)	106 (73)	38 (26)	0 (0)	0 (0)	18 (12)	11 (8)	61 (42)	117 (80)
Eulipotyphla	450	397 (88)	0 (0)	53 (12)	0 (0)	4 (1)	447 (99)	2 (0)	7 (2)	0 (0)
Hyracoidea	5	0 (0)	5 (100)	0 (0)	0 (0)	0 (0)	0 (0)	0 (0)	3 (60)	5 (100)
Lagomorpha	93	0 (0)	91 (98)	1 (1)	1 (1)	0 (0)	0 (0)	3 (3)	3 (3)	77 (83)
Macroscelidea	17	5 (29)	0 (0)	12 (71)	0 (0)	0 (0)	17 (100)	0 (0)	0 (0)	0 (0)
Microbiotheria	1	1 (100)	0 (0)	0 (0)	0 (0)	0 (0)	1 (100)	0 (0)	0 (0)	0 (0)
Monotremata	5	5 (100)	0 (0)	0 (0)	0 (0)	0 (0)	5 (100)	0 (0)	0 (0)	0 (0)
Notoryctemorphia	2	1 (50)	0 (0)	1 (50)	0 (0)	0 (0)	2 (100)	0 (0)	0 (0)	0 (0)
Paucituberculata	6	2 (33)	0 (0)	2 (33)	2 (33)	0 (0)	6 (100)	0 (0)	0 (0)	0 (0)
Peramelemorphia	22	4 (18)	4 (18)	11 (50)	3 (14)	0 (0)	14 (64)	1 (5)	7 (32)	0 (0)
Perissodactyla	16	0 (0)	16 (100)	0 (0)	0 (0)	0 (0)	0 (0)	0 (0)	6 (38)	15 (94)
Pholidota	8	8 (100)	0 (0)	0 (0)	0 (0)	0 (0)	8 (100)	0 (0)	0 (0)	0 (0)
Pilosa	10	4 (40)	6 (60)	0 (0)	0 (0)	0 (0)	4 (40)	0 (0)	2 (20)	5 (50)
Primates	415	13 (3)	204 (49)	198 (48)	0 (0)	4 (1)	150 (36)	58 (14)	272 (66)	181 (44)
Proboscidea	2	0 (0)	2 (100)	0 (0)	0 (0)	0 (0)	0 (0)	0 (0)	2 (100)	2 (100)
Rodentia	2256	101 (4)	1015 (45)	1133 (50)	7 (0)	4 (0)	912 (40)	1074 (48)	835 (37)	832 (37)
Scandentia	20	2 (10)	0 (0)	18 (90)	0 (0)	1 (5)	20 (100)	0 (0)	2 (10)	0 (0)
Tubulidentata	1	1 (100)	0 (0)	0 (0)	0 (0)	0 (0)	1 (100)	0 (0)	0 (0)	0 (0)
Total	5364	1637 (31)	1926 (36)	1788 (33)	13 (0)	209 (4)	2821 (53)	1204 (22)	1692 (32)	1473 (27)

### Spatial visualization

**Figure 7:**
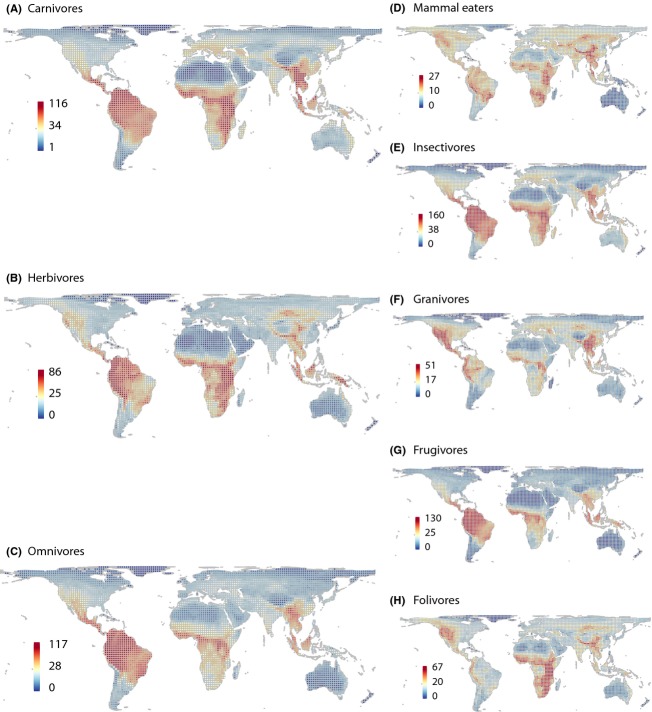
Global species richness maps of trophic levels (A–C) and dietary guilds (D–H). For definition of trophic levels and dietary guilds see Table 5. The grids are in Behrmann projection (a cylindrical equal-area projection) with a resolution of 2° equivalents. Color ramps are in quantile classification, numbers beside color ramps refer to species richness. Grid cells with less than 50% land cover as well as those covering Antarctica are not included.

Peaks in species richness of trophic levels showed a surprising spatial overlap across the world (Fig. 7A–C). This indicated that the build-up of species richness in different trophic levels is possibly governed by similar drivers. In contrast to coarse trophic levels, dietary guilds showed more spatial heterogeneity in species richness at a global scale (Fig. 7D–H). For instance, mammal eaters, granivores, and folivores appeared to be particularly species-rich in mountain ranges such as the Andes, Himalayas, East African mountains, and the mountainous west of the USA (Fig. 7D, F, H). In contrast, species richness of frugivores and insectivores additionally peaked in lowland tropical rainforests on all continents (Fig. 7E, G).

**Figure 8:**
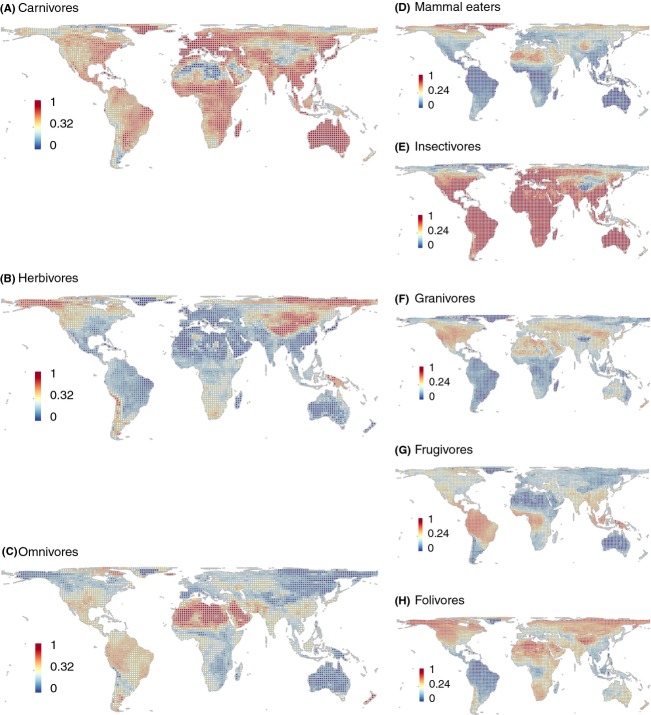
Proportions of trophic levels (A–C) and dietary guilds (D–H). For definition of trophic levels and dietary guilds see Table 5. The grids are in Behrmann projection (a cylindrical equal-area projection) with a resolution of 2° equivalents. Color ramps are in quantile classification. Grid cells with less than 50% land cover as well as those covering Antarctica are not included.

Beyond species richness, we also spatially visualized the proportions of each trophic level and dietary guild (Fig. 8). For trophic levels, carnivores showed high proportions in most parts of the world (Fig. 8A), whereas herbivores dominated mostly at high latitudes (Fig. 8B). Omnivores seemed to be proportionally overrepresented in the Saharan desert region (Fig. 8C), but this region is generally species poor. Proportional maps for dietary guilds showed that insectivores had high proportions throughout the world (Fig. 8E), frugivores mostly had high proportions around the equator (Fig. 8G), and mammal eaters, granivores, and folivores were well represented outside the tropical belt (Fig. 8D, F, H).

## Discussion

By digitizing, extrapolating, and validating diet preferences of terrestrial mammals worldwide, we compiled a comprehensive and unique, cladewide trait dataset (MammalDIET) relevant for macroecological and macroevolutionary analyses. In contrast to previous datasets that have been made available to the public (Jones et al. 2009; Price et al. 2012; Cantalapiedra et al. 2014), MammalDIET allows a finer dietary guild classification and a broader taxonomic coverage. This was achieved by a combination of original and extrapolated data, thus providing species-level diet estimates for >99% of all terrestrial mammals. Results from the internal and external validation steps confirmed the use of several diet categories as reliable information for subsequent classification of species into trophic levels and dietary guilds. The methodological approach used here (summarized in Fig. 2) could also be applied more widely when constructing global databases of species-specific traits.

Digitalization of available trait data represents an important step in the compilation of macroecological trait datasets. During this process, errors can occur, for example, when written text descriptions are converted into (semi)quantitative data. We used a calibration step with 20 randomly selected species before entering the data to ensure that diet information was digitized in the most consistent way among multiple data recorders. Furthermore, we tested the error rate due to data entry via multiple data recorders using 120 randomly selected species. This revealed that converting written diet descriptions from textbooks into (semi)quantitative diet ranks was not particularly prone to errors. We found that most diet ranks were entered in the same way by multiple recorders, with an accuracy of almost 95%. Nevertheless, some diet categories such as other plant material (“Other”) had a lower success rate (86%) which demonstrates a larger uncertainty in the assigned importance score for such unspecific categories. We emphasize that initial calibrations and subsequent data quality tests were valuable steps to avoid discrepancies in data entries and to maintain the consistency of data entry by multiple data recorders. Other authors of mammalian diet datasets (e.g., Price et al. 2012) also verbally report such cross-validations of scoring by multiple recorders although quantitative assessments are usually not provided. We therefore suggest that explicit guidelines for how to convert diet descriptions into ranked importance scores are needed when many recorders are involved in building up macroecological trait datasets (Jones et al. 2009).

To accommodate the lack of species-level traits in sparse datasets, an extrapolation or prediction of missing trait data based on non-missing entries from other taxonomic or phylogenetic levels might often be the only way to compile macroecological trait datasets with a global coverage (Shan et al. 2012). Our hierarchical extrapolation procedure allowed to fill-in gaps of diet information when species-level information was not available from the two original data sources. For some taxonomic groups (e.g., Rodentia, Eulipotyphla), the missing data reflect the limited diet knowledge at the species level. This became evident in the external validation which showed that for many extrapolated species additional species-level diet data were not available, even not from the most comprehensive regional data sources (Butynski et al. 2013; Happold 2013; Happold and Happold 2013; Kingdon and Hoffmann 2013a,b2013b; Kingdon et al. 2013). For instance, for Eulipotyphla (here mostly represented by shrews of the genus *Crocidura* in the family Soricidae) and Rodentia (various mice genera in the family Muridae), the external validation dataset based on the Mammals of Africa did not provide species-level diet information for 65% and 49% of the species, respectively. Nevertheless, we acknowledge that more species-level diet data could be extracted from additional data sources for some of the species which currently have extrapolated diets in MammalDIET. In such cases, MammalDIET could serve as a baseline source for adding additional data and the data coverage for such species could then be improved.

Extrapolation will be most reliable if taxa show a high level of phylogenetic conservatism in their diets. An excellent example of such diet conservatism is the microbats (suborder Microchiroptera in the order Chiroptera) which nearly all feed exclusively — as aerial insectivores — on insects and arthropods. For such groups, extrapolating diet knowledge from suborder, family or genus level will be unproblematic. Other mammal groups also show a high predictability for specific diet categories (Fig. 6). For instance, almost all species in the order Eulipotyphla feed primarily on invertebrates, including the shrews (family Soricidae) and the moles, shrew moles, and desmans (family Talpidae). Categorizing these species as insectivores (as defined in Table 5) is unproblematic even if diet knowledge at the species-level is absent. Nevertheless, several other diet categories are used by only a subset of Eulipotyphla species and an extrapolation in these cases is then less reliable (Fig. 6C). This similarly applies to primates (Primates) and even-toed ungulates (within Cetartiodactyla) which primarily feed on plant material (high phylogenetic conservatism and good predictability), but the specific type of plant material (fruits, seeds, leaves) can vary among species, genera and families, making predictions more difficult (Fig. 6A, B). More generally, the use of specific diet categories can be quite heterogeneous among species within several mammal orders, families and genera. Thus, uncertainty in extrapolating diet information across taxonomic levels depends on the level of diet generalization within taxonomic groups (Fig. 5) and on the hierarchical position of the diet categories (Fig. 3). For instance, some families in the order Rodentia (e.g., Cricetidae, to which true hamsters, voles, lemmings, and New World rats and mice belong) contain insectivorous, herbivorous and omnivorous species, and extrapolations from one species to another or from genus and family level will be less reliable. More generally, predictions across taxonomic levels will be more difficult if species within a certain taxonomic level (e.g., genus) use a large number of diet categories at low hierarchical levels. Despite this, our validations showed a surprisingly good predictive ability across the mammal clade for several diet categories, including the “Animal”, “Mammal”, “Invertebrate”, “Plant”, “Seed”, “Fruit”, and “Leaf” categories.

Compared with previously published datasets, MammalDIET represents an improved classification of dietary guilds in terrestrial mammals worldwide because the diet data is more detailed and provided in a quantitative format that facilitates customized diet reclassifications. For instance, Price et al. (2012) assembled coarse mammalian diet data and classified species into three trophic levels (carnivores, omnivores, herbivores), covering only approximately one-third of the mammals (*n* = 1530 species). Jones et al. (2009) recorded eight diet categories and classified mammals into three trophic levels (carnivores, omnivores, herbivores), but only for around 40% of the species. Jetz et al. (2009) compiled diet data for >90% of the mammal species, but only distinguished two trophic levels (primary and secondary consumers), and the data were not made publicly available. MammalDIET provides data for 16 diet categories that can be combined in many ways to generate any kind of customized dietary guilds. This enables a much more refined classification of dietary guilds than previously possible, and researchers are free to define diet guilds tailored to the question they are investigating. Our validation results further support previously applied classifications (e.g., Sandom et al. 2013) and suggest that results using 2–3 trophic levels based on similar data (Jetz et al. 2009; Price et al. 2012) should be relatively robust and reliable.

Until now, no global dataset on detailed mammalian diets and high species coverage has been publicly available. MammalDIET therefore constitutes a new global resource to enable macroecological and macroevolutionary analyses in mammals. For instance, MammalDIET could be used for phylogenetic approaches to study diversification, for example, to fit character-dependent diversification models and to estimate how diversification rates depend on mammalian diets (Morlon 2014). Previous diversification analyses were restricted to subsets of mammal species and to three trophic levels (Price et al. 2012; Cantalapiedra et al. 2014). Furthermore, macroecological analyses on global variation in species richness, co-occurrence and assemblage structure in combination with mammalian traits are now possible, for example, similar to those done for birds (Kissling et al. 2009, 2012; Barnagaud et al. 2014). A first analysis using a preliminary version of MammalDIET investigated global variation in mammalian predators and prey (Sandom et al. 2013), suggesting that trophic interactions can be important drivers of large-scale species richness gradients in combination with environmental effects. MammalDIET can further be used to explore the functional roles and functional diversity of mammals in ecosystems worldwide (Safi et al. 2011; Mazel et al. 2014), or to test whether body size, geography and diet interact in determining extinction risk (Smith et al. 2003; Cardillo et al. 2005; Fritz et al. 2009). We here provide MammalDIET to foster new analyses on mammal diversity along broad-scale (e.g., regional, continental or global) environmental gradients. We expect that statistical results at macroecological scales using the validated diet categories will be relatively robust, even though many species have diet information that is extrapolated from the genus level. We further suggest that MammalDIET could serve as a baseline to compile additional and more detailed diet data, for example, for specific subsets of species, certain regions, or when focussing on specific dietary guilds. Some diet categories such as “Herptile” (reptiles and amphibians), “Invertebrate” (incl. social and nonsocial insects, arthropods, aquatic and terrestrial invertebrates), or “Other” (incl. buds, flowers, gum, fungi, etc.) could even be refined in the future.

## Conclusions

The compilation of macroecological trait datasets such as MammalDIET is challenging and requires several methodological steps, from digitizing accessible information to extrapolating missing data and validating extrapolation procedures. The approach illustrated here provides an example to fill-in data gaps in mammalian trait information and could be applicable more widely to other traits and taxa. Due to large knowledge gaps on traits of species-rich clades, we suggest that a comprehensive effort into the compilation and prediction of traits is needed to significantly advance macroecological and macroevolutionary research. Fundamental to this effort will be a deeper understanding of phylogenetic conservatism in traits, that is, when it matters and how it varies across taxonomic and phylogenetic scales.

## Supporting Information

**Appendix Table S1.** MammalDIET metadata. **Appendix Table S2.** Mammal orders and families and their summary information for extrapolating data on diet preferences from other species or other taxonomic levels (genus, family) to species for which species-level information was missing. **Appendix Table S3.** Number of species within each mammal family and the percentage of the species assigned to three trophic levels (carnivores, herbivores, and omnivores) and five dietary guilds (mammal eaters, insectivores, granivores, frugivores and folivores).
