# Supplementary material for: Establishing macroecological trait datasets: digitalization, extrapolation, and validation of diet preferences in terrestrial mammals worldwide
Source: Ecol Evol. 2014 Jun 16;4(14):2913–30. doi: 10.1002/ece3.1136 (PMC4130448; doi:10.1002/ece3.1136)
Supplement: Supplementary file 1 [file ece30004-2913-SD1.doc]

**Appendix Table S1:** MammalDIET metadata.

| **Variable name** | **Variable definition** | **Units** | **Storage type** | **Variable codes and definitions** | **Missing value codes** |
| --- | --- | --- | --- | --- | --- |
| TaxonID | Unique numeric code for each species, starting with 1 and ending with 5364. | Not available | Integer | Not available |  |
| Order | The mammal order name of each species, following the IUCN taxonomy . | Not available | Character | Not available |  |
| Family | The mammal family name of each species, following the IUCN taxonomy . | Not available | Character | Not available |  |
| Genus | The mammal genus name of each species, following the IUCN taxonomy . | Not available | Character | Not available |  |
| Species | The mammal species name, following the IUCN taxonomy . | Not available | Character | Not available |  |
| Animal | Whether a mammal species eats animals (rank 1–3) or not (rank 0). | Not available | Integer | 0 = does not eat animals; 1 = animals as primary food item; 2 = animals as secondary food item; 3 = animals as occasional food item. | NA |
| Vertebrate | Whether a mammal species eats vertebrates (1-3) or not (0). | Not available | Integer | 0 = does not eat vertebrates; 1 = vertebrates as primary food item; 2 = vertebrates as secondary food item; 3 = vertebrates as occasional food item. | NA |
| Mammal | Whether a mammal species eats other mammals (rank 1–3) or not (rank 0). | Not available | Integer | 0 = does not eat mammals; 1 = mammals as primary food item; 2 = mammals as secondary food item; 3 = mammals as occasional food item. | NA |
| Bird | Whether a mammal species eats birds (rank 1–3) or not (rank 0). | Not available | Integer | 0 = does not eat birds; 1 = birds as primary food item; 2 = birds as secondary food item; 3 = birds as occasional food item. | NA |
| Herptile | Whether a mammal species eats herptiles (amphibians or reptiles) (rank 1–3) or not (rank 0). | Not available | Integer | 0 = does not eat herptiles; 1 = herptiles as primary food item; 2 = herptiles as secondary food item; 3 = herptiles as occasional food item. | NA |
| Fish | Whether a mammal species eats fish (rank 1–3) or not (rank 0). | Not available | Integer | 0 = does not eat fish; 1 = fish as primary food item; 2 = fish as secondary food item; 3 = fish as occasional food item. | NA |
| Invertebrate | Whether a mammal species eats invertebrates (rank 1–3) or not (rank 0). | Not available | Integer | 0 = does not eat invertebrates; 1 = invertebrates as primary food item; 2 = invertebrates as secondary food item; 3 = invertebrates as occasional food item. | NA |
| Plant | Whether a mammal species eats plants (rank 1–3) or not (rank 0). | Not available | Integer | 0 = does not eat plants; 1 = plants as primary food item; 2 = plants as secondary food item; 3 = plants as occasional food item. | NA |
| Seed | Whether a mammal species eats seeds (rank 1–3) or not (rank 0). | Not available | Integer | 0 = does not eat seeds; 1 = seeds as primary food item; 2 = seeds as secondary food item; 3 = seeds as occasional food item. | NA |
| Fruit | Whether a mammal species eats fleshy fruits (rank 1–3) or not (rank 0). | Not available | Integer | 0 = does not eat fleshy fruits; 1 = fleshy fruits as primary food item; 2 = fleshy fruits as secondary food item; 3 = fleshy fruits as occasional food item. | NA |
| Nectar | Whether a mammal species eats nectar (rank 1–3) or not (rank 0). | Not available | Integer | 0 = does not eat nectar; 1 = nectar as primary food item; 2 = nectar as secondary food item; 3 = nectar as occasional food item. | NA |
| Root | Whether a mammal species eats roots (rank 1–3) or not (rank 0). | Not available | Integer | 0 = does not eat roots; 1 = roots as primary food item; 2 = roots as secondary food item; 3 = roots as occasional food item. | NA |
| Leaf | Whether a mammal species eats leaves (rank 1–3) or not (rank 0). | Not available | Integer | 0 = does not eat leaves; 1 = leaves as primary food item; 2 = leaves as secondary food item; 3 = leaves as occasional food item. | NA |
| Woody | Whether a mammal species eats leaves of woody plants (rank 1–3) or not (rank 0). Allows the definition of browsers (as opposed to grazers). | Not available | Integer | 0 = does not eat leaves of woody plants; 1 = leaves of woody plants as primary food item; 2 = leaves of woody plants as secondary food item; 3 = leaves of woody plants as occasional food item. | NA |
| Herbaceous | Whether a mammal species eats leaves of herbaceous plants (rank 1–3) or not (rank 0). Allows the definition of grazers (as opposed to browsers). | Not available | Integer | 0 = does not eat leaves of herbaceous plants; 1 = leaves of herbaceous plants as primary food item; 2 = leaves of herbaceous plants as secondary food item; 3 = leaves of herbaceous plants as occasional food item. | NA |
| Other | Whether a mammal species eats other plant material (rank 1–3) or not (rank 0). Other plant material refers to plant food items that are not covered by the other plant categories (e.g., buds, flowers, pollen, and gum). It also includes fungi and lichens. | Not available | Integer | 0 = does not eat other plant material; 1 = other plant material as primary food item; 2 = other plant material as secondary food item; 3 = other plant material as occasional food item. | NA |
| TaxonomicNote | Information on synonyms (species, genus, family), i.e., differences in taxonomy between and . | Not available | Character | Not available |  |
| FillCode | Data extrapolation method for species without species-level diet information in . | Not available | Floating point | 0 = species with species-level diet information in Nowak (1999); 1 = diet data were filled from available genus level information; 2.1 = diet data were filled from one species within the same genus where diet information was available; 2.2 = diet information was filled from other species within the same genus if all species had equal information, otherwise data were assigned as not available (NA); 3 = diet data were filled from available diet information at the family level. | NA |
| TrophicLevel | A classification of species into three trophic levels. | Not available | Character | Carnivore (predominantly eating animals); Herbivore (predominantly eating plant material); Omnivore (feeding on both animals and plants); NotAssigned (species which do not fit in any of the previous categories). |  |
| MammalEater | Species with mammals as an important diet item | Not available | Integer | 1 = species with rank 1 in diet category ‘Mammal’; 0 = species without rank 1 in ‘Mammal’ |  |
| Insectivore | Species with invertebrates as an important diet item | Not available | Integer | 1 = species with rank 1 in diet category ‘Invertebrate’; 0 = species without rank 1 in ‘Invertebrate’ |  |
| Frugivore | Species with fruits as an important diet item | Not available | Integer | 1 = species with rank 1 in diet category ‘Fruit’; 0 = species without rank 1 in ‘Fruit’ |  |
| Granivore | Species with seeds as an important diet item | Not available | Integer | 1 = species with rank 1 in diet category ‘Seed’; 0 = species without rank 1 in ‘Seed’ |  |
| Folivore | Species with leaves as an important diet item | Not available | Integer | 1 = species with rank 1 in diet category ‘Leaf’; 0 = species without rank 1 in ‘Leaf’ |  |
| DataSource | Whether data come from , , or whether they have been extrapolated. | Not available | Character | Nowak = data derived from ; IUCN = data derived from ; Extrapolated = data extrapolated from genus, other species within the same genus, or from family (see column ‘FillCode’) |  |

**Appendix Table S2:** Mammal orders and families and their summary information for extrapolating data on diet preferences from other species or other taxonomic levels (genus, family) to species for which species-level information was missing. Diet information was digitized from the literature and available for 2033 species at the species level (FillCode = 0). For the other species (*n* = 3331 species), diet data were first filled from the genus level (FillCode = 1, *n* = 2556 species), then from one other congeneric species (FillCode = 2.1, *n* = 266 species) or from >1 species within the same genus (FillCode = 2.2, *n* = 71 species), and finally from the family level (FillCode = 3, *n* = 436 species). See text for details and evaluation of extrapolation procedure.

|  |  |  |  |  |  |  |  |
| --- | --- | --- | --- | --- | --- | --- | --- |
|  |  |  | Number of species (percentage) in FillCode | | | | |
|  |  |  |  |  |  |  |  |
|  |  |  |  |  |  |  |  |
| Order | Family | Total species | 0 | 1 | 2.1 | 2.2 | 3 |
|  |  |  |  |  |  |  |  |
|  |  |  |  |  |  |  |  |
| Afrosoricida | Chrysochloridae | 21 | 5 (24) | 14 (67) | 2 (10) | 0 (0) | 0 (0) |
| Afrosoricida | Tenrecidae | 33 | 10 (30) | 22 (67) | 1 (3) | 0 (0) | 0 (0) |
| Carnivora | Ailuridae | 1 | 1 (100) | 0 (0) | 0 (0) | 0 (0) | 0 (0) |
| Carnivora | Canidae | 36 | 31 (86) | 5 (14) | 0 (0) | 0 (0) | 0 (0) |
| Carnivora | Eupleridae | 9 | 7 (78) | 0 (0) | 2 (22) | 0 (0) | 0 (0) |
| Carnivora | Felidae | 36 | 34 (94) | 0 (0) | 1 (3) | 1 (3) | 0 (0) |
| Carnivora | Herpestidae | 34 | 24 (71) | 10 (29) | 0 (0) | 0 (0) | 0 (0) |
| Carnivora | Hyaenidae | 4 | 4 (100) | 0 (0) | 0 (0) | 0 (0) | 0 (0) |
| Carnivora | Mephitidae | 12 | 12 (100) | 0 (0) | 0 (0) | 0 (0) | 0 (0) |
| Carnivora | Mustelidae | 59 | 49 (83) | 4 (7) | 0 (0) | 6 (10) | 0 (0) |
| Carnivora | Nandiniidae | 1 | 1 (100) | 0 (0) | 0 (0) | 0 (0) | 0 (0) |
| Carnivora | Prionodontidae | 2 | 2 (100) | 0 (0) | 0 (0) | 0 (0) | 0 (0) |
| Carnivora | Procyonidae | 14 | 13 (93) | 1 (7) | 0 (0) | 0 (0) | 0 (0) |
| Carnivora | Ursidae | 8 | 8 (100) | 0 (0) | 0 (0) | 0 (0) | 0 (0) |
| Carnivora | Viverridae | 33 | 17 (52) | 15 (45) | 1 (3) | 0 (0) | 0 (0) |
| Cetartiodactyla | Antilocapridae | 1 | 1 (100) | 0 (0) | 0 (0) | 0 (0) | 0 (0) |
| Cetartiodactyla | Bovidae | 140 | 93 (66) | 38 (27) | 7 (5) | 2 (1) | 0 (0) |
| Cetartiodactyla | Camelidae | 3 | 3 (100) | 0 (0) | 0 (0) | 0 (0) | 0 (0) |
| Cetartiodactyla | Cervidae | 55 | 35 (64) | 17 (31) | 2 (4) | 1 (2) | 0 (0) |
| Cetartiodactyla | Giraffidae | 2 | 2 (100) | 0 (0) | 0 (0) | 0 (0) | 0 (0) |
| Cetartiodactyla | Hippopotamidae | 4 | 4 (100) | 0 (0) | 0 (0) | 0 (0) | 0 (0) |
| Cetartiodactyla | Moschidae | 7 | 4 (57) | 0 (0) | 0 (0) | 0 (0) | 3 (43) |
| Cetartiodactyla | Suidae | 18 | 8 (44) | 10 (56) | 0 (0) | 0 (0) | 0 (0) |
| Cetartiodactyla | Tayassuidae | 4 | 4 (100) | 0 (0) | 0 (0) | 0 (0) | 0 (0) |
| Cetartiodactyla | Tragulidae | 10 | 6 (60) | 0 (0) | 4 (40) | 0 (0) | 0 (0) |
| Chiroptera | Craseonycteridae | 1 | 1 (100) | 0 (0) | 0 (0) | 0 (0) | 0 (0) |
| Chiroptera | Emballonuridae | 52 | 22 (42) | 29 (56) | 0 (0) | 0 (0) | 1 (2) |
| Chiroptera | Furipteridae | 2 | 2 (100) | 0 (0) | 0 (0) | 0 (0) | 0 (0) |
| Chiroptera | Hipposideridae | 84 | 16 (19) | 58 (69) | 0 (0) | 0 (0) | 10 (12) |
| Chiroptera | Megadermatidae | 5 | 4 (80) | 1 (20) | 0 (0) | 0 (0) | 0 (0) |
| Chiroptera | Molossidae | 100 | 47 (47) | 13 (13) | 4 (4) | 21 (21) | 15 (15) |
| Chiroptera | Mormoopidae | 9 | 8 (89) | 0 (0) | 0 (0) | 1 (11) | 0 (0) |
| Chiroptera | Mystacinidae | 2 | 1 (50) | 1 (50) | 0 (0) | 0 (0) | 0 (0) |
| Chiroptera | Myzopodidae | 2 | 1 (50) | 1 (50) | 0 (0) | 0 (0) | 0 (0) |
| Chiroptera | Natalidae | 11 | 8 (73) | 3 (27) | 0 (0) | 0 (0) | 0 (0) |
| Chiroptera | Noctilionidae | 2 | 2 (100) | 0 (0) | 0 (0) | 0 (0) | 0 (0) |
| Chiroptera | Nycteridae | 16 | 1 (6) | 15 (94) | 0 (0) | 0 (0) | 0 (0) |
| Chiroptera | Phyllostomidae | 174 | 139 (80) | 32 (18) | 0 (0) | 2 (1) | 1 (1) |
| Chiroptera | Pteropodidae | 187 | 59 (32) | 97 (52) | 13 (7) | 2 (1) | 16 (9) |
| Chiroptera | Rhinolophidae | 74 | 10 (14) | 64 (86) | 0 (0) | 0 (0) | 0 (0) |
| Chiroptera | Rhinopomatidae | 4 | 1 (25) | 3 (75) | 0 (0) | 0 (0) | 0 (0) |
| Chiroptera | Thyropteridae | 4 | 3 (75) | 1 (25) | 0 (0) | 0 (0) | 0 (0) |
| Chiroptera | Vespertilionidae | 421 | 124 (29) | 237 (56) | 4 (1) | 0 (0) | 56 (13) |
| Cingulata | Dasypodidae | 21 | 4 (19) | 17 (81) | 0 (0) | 0 (0) | 0 (0) |
| Dasyuromorphia | Dasyuridae | 72 | 18 (25) | 52 (72) | 0 (0) | 0 (0) | 2 (3) |
| Dasyuromorphia | Myrmecobiidae | 1 | 1 (100) | 0 (0) | 0 (0) | 0 (0) | 0 (0) |
| Dasyuromorphia | Thylacinidae | 1 | 1 (100) | 0 (0) | 0 (0) | 0 (0) | 0 (0) |
| Dermoptera | Cynocephalidae | 2 | 1 (50) | 0 (0) | 1 (50) | 0 (0) | 0 (0) |
| Didelphimorphia | Didelphidae | 95 | 39 (41) | 50 (53) | 6 (6) | 0 (0) | 0 (0) |
| Diprotodontia | Acrobatidae | 2 | 2 (100) | 0 (0) | 0 (0) | 0 (0) | 0 (0) |
| Diprotodontia | Burramyidae | 5 | 1 (20) | 4 (80) | 0 (0) | 0 (0) | 0 (0) |
| Diprotodontia | Hypsiprymnodontidae | 1 | 1 (100) | 0 (0) | 0 (0) | 0 (0) | 0 (0) |
| Diprotodontia | Macropodidae | 67 | 9 (13) | 57 (85) | 1 (1) | 0 (0) | 0 (0) |
| Diprotodontia | Petauridae | 11 | 5 (45) | 6 (55) | 0 (0) | 0 (0) | 0 (0) |
| Diprotodontia | Phalangeridae | 26 | 6 (23) | 17 (65) | 3 (12) | 0 (0) | 0 (0) |
| Diprotodontia | Phascolarctidae | 1 | 1 (100) | 0 (0) | 0 (0) | 0 (0) | 0 (0) |
| Diprotodontia | Potoroidae | 11 | 6 (55) | 3 (27) | 2 (18) | 0 (0) | 0 (0) |
| Diprotodontia | Pseudocheiridae | 18 | 7 (39) | 11 (61) | 0 (0) | 0 (0) | 0 (0) |
| Diprotodontia | Tarsipedidae | 1 | 1 (100) | 0 (0) | 0 (0) | 0 (0) | 0 (0) |
| Diprotodontia | Vombatidae | 3 | 2 (67) | 1 (33) | 0 (0) | 0 (0) | 0 (0) |
| Eulipotyphla | Erinaceidae | 24 | 8 (33) | 12 (50) | 2 (8) | 0 (0) | 2 (8) |
| Eulipotyphla | Nesophontidae | 6 | 5 (83) | 1 (17) | 0 (0) | 0 (0) | 0 (0) |
| Eulipotyphla | Solenodontidae | 3 | 2 (67) | 1 (33) | 0 (0) | 0 (0) | 0 (0) |
| Eulipotyphla | Soricidae | 376 | 77 (20) | 231 (61) | 38 (10) | 0 (0) | 30 (8) |
| Eulipotyphla | Talpidae | 41 | 19 (46) | 8 (20) | 5 (12) | 0 (0) | 9 (22) |
| Hyracoidea | Procaviidae | 5 | 2 (40) | 3 (60) | 0 (0) | 0 (0) | 0 (0) |
| Lagomorpha | Leporidae | 62 | 30 (48) | 31 (50) | 1 (2) | 0 (0) | 0 (0) |
| Lagomorpha | Ochotonidae | 30 | 16 (53) | 14 (47) | 0 (0) | 0 (0) | 0 (0) |
| Lagomorpha | Prolagidae | 1 | 0 (0) | 0 (0) | 0 (0) | 0 (0) | 0 (0) |
| Macroscelidea | Macroscelididae | 17 | 4 (24) | 13 (76) | 0 (0) | 0 (0) | 0 (0) |
| Microbiotheria | Microbiotheriidae | 1 | 1 (100) | 0 (0) | 0 (0) | 0 (0) | 0 (0) |
| Monotremata | Ornithorhynchidae | 1 | 1 (100) | 0 (0) | 0 (0) | 0 (0) | 0 (0) |
| Monotremata | Tachyglossidae | 4 | 4 (100) | 0 (0) | 0 (0) | 0 (0) | 0 (0) |
| Notoryctemorphia | Notoryctidae | 2 | 1 (50) | 1 (50) | 0 (0) | 0 (0) | 0 (0) |
| Paucituberculata | Caenolestidae | 6 | 4 (67) | 0 (0) | 0 (0) | 2 (33) | 0 (0) |
| Peramelemorphia | Chaeropodidae | 1 | 0 (0) | 1 (100) | 0 (0) | 0 (0) | 0 (0) |
| Peramelemorphia | Peramelidae | 19 | 5 (26) | 9 (47) | 2 (11) | 3 (16) | 0 (0) |
| Peramelemorphia | Thylacomyidae | 2 | 1 (50) | 1 (50) | 0 (0) | 0 (0) | 0 (0) |
| Perissodactyla | Equidae | 7 | 7 (100) | 0 (0) | 0 (0) | 0 (0) | 0 (0) |
| Perissodactyla | Rhinocerotidae | 5 | 5 (100) | 0 (0) | 0 (0) | 0 (0) | 0 (0) |
| Perissodactyla | Tapiridae | 4 | 1 (25) | 3 (75) | 0 (0) | 0 (0) | 0 (0) |
| Pholidota | Manidae | 8 | 7 (88) | 0 (0) | 0 (0) | 0 (0) | 1 (12) |
| Pilosa | Bradypodidae | 4 | 2 (50) | 2 (50) | 0 (0) | 0 (0) | 0 (0) |
| Pilosa | Cyclopedidae | 1 | 0 (0) | 1 (100) | 0 (0) | 0 (0) | 0 (0) |
| Pilosa | Megalonychidae | 2 | 1 (50) | 1 (50) | 0 (0) | 0 (0) | 0 (0) |
| Pilosa | Myrmecophagidae | 3 | 1 (33) | 1 (33) | 0 (0) | 0 (0) | 1 (33) |
| Primates | Aotidae | 11 | 9 (82) | 2 (18) | 0 (0) | 0 (0) | 0 (0) |
| Primates | Atelidae | 28 | 25 (89) | 3 (11) | 0 (0) | 0 (0) | 0 (0) |
| Primates | Callitrichidae | 42 | 39 (93) | 3 (7) | 0 (0) | 0 (0) | 0 (0) |
| Primates | Cebidae | 17 | 12 (71) | 5 (29) | 0 (0) | 0 (0) | 0 (0) |
| Primates | Cercopithecidae | 122 | 74 (61) | 47 (39) | 0 (0) | 1 (1) | 0 (0) |
| Primates | Cheirogaleidae | 29 | 1 (3) | 28 (97) | 0 (0) | 0 (0) | 0 (0) |
| Primates | Daubentoniidae | 1 | 1 (100) | 0 (0) | 0 (0) | 0 (0) | 0 (0) |
| Primates | Galagidae | 18 | 5 (28) | 0 (0) | 13 (72) | 0 (0) | 0 (0) |
| Primates | Hominidae | 7 | 7 (100) | 0 (0) | 0 (0) | 0 (0) | 0 (0) |
| Primates | Hylobatidae | 16 | 14 (88) | 2 (12) | 0 (0) | 0 (0) | 0 (0) |
| Primates | Indriidae | 18 | 3 (17) | 9 (50) | 6 (33) | 0 (0) | 0 (0) |
| Primates | Lemuridae | 20 | 10 (50) | 9 (45) | 1 (5) | 0 (0) | 0 (0) |
| Primates | Lepilemuridae | 24 | 0 (0) | 24 (100) | 0 (0) | 0 (0) | 0 (0) |
| Primates | Lorisidae | 10 | 8 (80) | 1 (10) | 1 (10) | 0 (0) | 0 (0) |
| Primates | Palaeopropithecidae | 1 | 0 (0) | 1 (100) | 0 (0) | 0 (0) | 0 (0) |
| Primates | Pitheciidae | 43 | 30 (70) | 13 (30) | 0 (0) | 0 (0) | 0 (0) |
| Primates | Tarsiidae | 8 | 5 (62) | 3 (38) | 0 (0) | 0 (0) | 0 (0) |
| Proboscidea | Elephantidae | 2 | 2 (100) | 0 (0) | 0 (0) | 0 (0) | 0 (0) |
| Rodentia | Abrocomidae | 10 | 3 (30) | 7 (70) | 0 (0) | 0 (0) | 0 (0) |
| Rodentia | Anomaluridae | 7 | 3 (43) | 0 (0) | 4 (57) | 0 (0) | 0 (0) |
| Rodentia | Aplodontiidae | 1 | 1 (100) | 0 (0) | 0 (0) | 0 (0) | 0 (0) |
| Rodentia | Bathyergidae | 15 | 2 (13) | 10 (67) | 0 (0) | 0 (0) | 3 (20) |
| Rodentia | Calomyscidae | 8 | 3 (38) | 5 (62) | 0 (0) | 0 (0) | 0 (0) |
| Rodentia | Capromyidae | 19 | 5 (26) | 2 (11) | 1 (5) | 0 (0) | 11 (58) |
| Rodentia | Castoridae | 2 | 0 (0) | 2 (100) | 0 (0) | 0 (0) | 0 (0) |
| Rodentia | Caviidae | 18 | 4 (22) | 13 (72) | 1 (6) | 0 (0) | 0 (0) |
| Rodentia | Chinchillidae | 7 | 3 (43) | 4 (57) | 0 (0) | 0 (0) | 0 (0) |
| Rodentia | Cricetidae | 698 | 218 (31) | 279 (40) | 80 (11) | 5 (1) | 116 (17) |
| Rodentia | Ctenodactylidae | 5 | 0 (0) | 0 (0) | 0 (0) | 0 (0) | 5 (100) |
| Rodentia | Ctenomyidae | 60 | 9 (15) | 51 (85) | 0 (0) | 0 (0) | 0 (0) |
| Rodentia | Cuniculidae | 2 | 1 (50) | 1 (50) | 0 (0) | 0 (0) | 0 (0) |
| Rodentia | Dasyproctidae | 13 | 6 (46) | 7 (54) | 0 (0) | 0 (0) | 0 (0) |
| Rodentia | Diatomyidae | 1 | 1 (100) | 0 (0) | 0 (0) | 0 (0) | 0 (0) |
| Rodentia | Dinomyidae | 1 | 1 (100) | 0 (0) | 0 (0) | 0 (0) | 0 (0) |
| Rodentia | Dipodidae | 50 | 37 (74) | 13 (26) | 0 (0) | 0 (0) | 0 (0) |
| Rodentia | Echimyidae | 89 | 20 (22) | 31 (35) | 6 (7) | 0 (0) | 32 (36) |
| Rodentia | Erethizontidae | 18 | 13 (72) | 4 (22) | 0 (0) | 0 (0) | 0 (0) |
| Rodentia | Geomyidae | 39 | 15 (38) | 21 (54) | 0 (0) | 0 (0) | 3 (8) |
| Rodentia | Gliridae | 28 | 7 (25) | 20 (71) | 0 (0) | 0 (0) | 1 (4) |
| Rodentia | Heteromyidae | 62 | 34 (55) | 22 (35) | 0 (0) | 6 (10) | 0 (0) |
| Rodentia | Hystricidae | 11 | 2 (18) | 9 (82) | 0 (0) | 0 (0) | 0 (0) |
| Rodentia | Muridae | 711 | 129 (18) | 470 (66) | 47 (7) | 6 (1) | 59 (8) |
| Rodentia | Myocastoridae | 1 | 1 (100) | 0 (0) | 0 (0) | 0 (0) | 0 (0) |
| Rodentia | Nesomyidae | 60 | 1 (2) | 20 (33) | 0 (0) | 0 (0) | 39 (65) |
| Rodentia | Octodontidae | 13 | 5 (38) | 7 (54) | 0 (0) | 0 (0) | 1 (8) |
| Rodentia | Pedetidae | 2 | 1 (50) | 0 (0) | 1 (50) | 0 (0) | 0 (0) |
| Rodentia | Petromuridae | 1 | 1 (100) | 0 (0) | 0 (0) | 0 (0) | 0 (0) |
| Rodentia | Platacanthomyidae | 2 | 2 (100) | 0 (0) | 0 (0) | 0 (0) | 0 (0) |
| Rodentia | Sciuridae | 279 | 107 (38) | 138 (49) | 3 (1) | 12 (4) | 19 (7) |
| Rodentia | Spalacidae | 21 | 12 (57) | 9 (43) | 0 (0) | 0 (0) | 0 (0) |
| Rodentia | Thryonomyidae | 2 | 0 (0) | 2 (100) | 0 (0) | 0 (0) | 0 (0) |
| Scandentia | Ptilocercidae | 1 | 1 (100) | 0 (0) | 0 (0) | 0 (0) | 0 (0) |
| Scandentia | Tupaiidae | 19 | 3 (16) | 16 (84) | 0 (0) | 0 (0) | 0 (0) |
| Tubulidentata | Orycteropodidae | 1 | 1 (100) | 0 (0) | 0 (0) | 0 (0) | 0 (0) |
| **TOTAL** |  | **5364** | **2033 (38)** | **2556 (47)** | **266 (5)** | **71 (1)** | **436 (8)** |
|  |  |  |  |  |  |  |  |

**Appendix Table S3:** Number of species within each mammal family and the percentage of the species assigned to three trophic levels (carnivores, herbivores, and omnivores) and five dietary guilds (mammal eaters, insectivores, granivores, frugivores and folivores). See Appendix Table S1 for a more detailed description of the trophic levels and dietary guilds.

|  |  |  |  | | | |  | |  | |  | |  | |  | |
| --- | --- | --- | --- | --- | --- | --- | --- | --- | --- | --- | --- | --- | --- | --- | --- | --- |
|  |  |  | Number of species (percentages) in each trophic level and dietary guild | | | | | | | | | | | | | |
|  |  |  |  | | | |  |  |  | |  | |  | |  | |
|  |  |  |  | | | |  |  |  | |  | |  | |  | |
|  |  | Total species number | Trophic levels | | | |  | Dietary guilds | | | | | | | | |
|  |  |  |  |  |  |  |  |  |  |  |  |  |  |  |  |  |
| Mammal order | Family |  | Carnivores | Insectivores | Herbivores | Omnivores |  | Mammal eaters | | Insectivores | | Granivores | | Frugivores | | Folivores |
|  |  |  |  |  |  |  |  |  | |  | |  | |  | |  |
|  |  |  |  |  |  |  |  |  | |  | |  | |  | |  |
| Afrosoricida | Chrysochloridae | 21 | 21 (100) | 0 (0) | 0 (0) | 0 (0) |  | 0 (0) | | 21 (100) | | 0 (0) | | 0 (0) | | 0 (0) |
| Afrosoricida | Tenrecidae | 33 | 32 (97) | 0 (0) | 1 (3) | 0 (0) |  | 1 (3) | | 33 (100) | | 0 (0) | | 0 (0) | | 0 (0) |
| Carnivora | Ailuridae | 1 | 0 (0) | 1 (100) | 0 (0) | 0 (0) |  | 0 (0) | | 0 (0) | | 1 (100) | | 1 (100) | | 1 (100) |
| Carnivora | Canidae | 36 | 14 (39) | 0 (0) | 22 (61) | 0 (0) |  | 27 (75) | | 19 (53) | | 2 (6) | | 12 (33) | | 1 (3) |
| Carnivora | Eupleridae | 9 | 7 (78) | 0 (0) | 2 (22) | 0 (0) |  | 5 (56) | | 4 (44) | | 0 (0) | | 1 (11) | | 0 (0) |
| Carnivora | Felidae | 36 | 35 (97) | 0 (0) | 1 (3) | 0 (0) |  | 32 (89) | | 0 (0) | | 0 (0) | | 0 (0) | | 0 (0) |
| Carnivora | Herpestidae | 34 | 17 (50) | 0 (0) | 17 (50) | 0 (0) |  | 12 (35) | | 30 (88) | | 0 (0) | | 11 (32) | | 0 (0) |
| Carnivora | Hyaenidae | 4 | 2 (50) | 0 (0) | 2 (50) | 0 (0) |  | 3 (75) | | 1 (25) | | 0 (0) | | 0 (0) | | 0 (0) |
| Carnivora | Mephitidae | 12 | 5 (42) | 0 (0) | 7 (58) | 0 (0) |  | 4 (33) | | 11 (92) | | 1 (8) | | 3 (25) | | 0 (0) |
| Carnivora | Mustelidae | 59 | 41 (69) | 0 (0) | 18 (31) | 0 (0) |  | 36 (61) | | 24 (41) | | 2 (3) | | 11 (19) | | 0 (0) |
| Carnivora | Nandiniidae | 1 | 0 (0) | 0 (0) | 1 (100) | 0 (0) |  | 0 (0) | | 0 (0) | | 0 (0) | | 1 (100) | | 0 (0) |
| Carnivora | Prionodontidae | 2 | 2 (100) | 0 (0) | 0 (0) | 0 (0) |  | 1 (50) | | 0 (0) | | 0 (0) | | 0 (0) | | 0 (0) |
| Carnivora | Procyonidae | 14 | 1 (7) | 0 (0) | 13 (93) | 0 (0) |  | 2 (14) | | 11 (79) | | 1 (7) | | 13 (93) | | 0 (0) |
| Carnivora | Ursidae | 8 | 0 (0) | 3 (38) | 5 (62) | 0 (0) |  | 2 (25) | | 3 (38) | | 0 (0) | | 3 (38) | | 4 (50) |
| Carnivora | Viverridae | 33 | 15 (45) | 1 (3) | 17 (52) | 0 (0) |  | 22 (67) | | 26 (79) | | 1 (3) | | 11 (33) | | 0 (0) |
| Cetartiodactyla | Antilocapridae | 1 | 0 (0) | 1 (100) | 0 (0) | 0 (0) |  | 0 (0) | | 0 (0) | | 0 (0) | | 0 (0) | | 1 (100) |
| Cetartiodactyla | Bovidae | 140 | 0 (0) | 125 (89) | 15 (11) | 0 (0) |  | 0 (0) | | 0 (0) | | 8 (6) | | 25 (18) | | 137 (98) |
| Cetartiodactyla | Camelidae | 3 | 0 (0) | 3 (100) | 0 (0) | 0 (0) |  | 0 (0) | | 0 (0) | | 0 (0) | | 0 (0) | | 2 (67) |
| Cetartiodactyla | Cervidae | 55 | 0 (0) | 55 (100) | 0 (0) | 0 (0) |  | 0 (0) | | 0 (0) | | 4 (7) | | 10 (18) | | 51 (93) |
| Cetartiodactyla | Giraffidae | 2 | 0 (0) | 2 (100) | 0 (0) | 0 (0) |  | 0 (0) | | 0 (0) | | 0 (0) | | 0 (0) | | 2 (100) |
| Cetartiodactyla | Hippopotamidae | 4 | 0 (0) | 4 (100) | 0 (0) | 0 (0) |  | 0 (0) | | 0 (0) | | 0 (0) | | 1 (25) | | 4 (100) |
| Cetartiodactyla | Moschidae | 7 | 0 (0) | 7 (100) | 0 (0) | 0 (0) |  | 0 (0) | | 0 (0) | | 0 (0) | | 0 (0) | | 7 (100) |
| Cetartiodactyla | Suidae | 18 | 0 (0) | 8 (44) | 10 (56) | 0 (0) |  | 0 (0) | | 8 (44) | | 8 (44) | | 16 (89) | | 12 (67) |
| Cetartiodactyla | Tayassuidae | 4 | 0 (0) | 1 (25) | 3 (75) | 0 (0) |  | 0 (0) | | 1 (25) | | 2 (50) | | 3 (75) | | 2 (50) |
| Cetartiodactyla | Tragulidae | 10 | 0 (0) | 10 (100) | 0 (0) | 0 (0) |  | 0 (0) | | 0 (0) | | 0 (0) | | 10 (100) | | 8 (80) |
| Chiroptera | Craseonycteridae | 1 | 1 (100) | 0 (0) | 0 (0) | 0 (0) |  | 0 (0) | | 1 (100) | | 0 (0) | | 0 (0) | | 0 (0) |
| Chiroptera | Emballonuridae | 52 | 52 (100) | 0 (0) | 0 (0) | 0 (0) |  | 0 (0) | | 52 (100) | | 0 (0) | | 0 (0) | | 0 (0) |
| Chiroptera | Furipteridae | 2 | 2 (100) | 0 (0) | 0 (0) | 0 (0) |  | 0 (0) | | 2 (100) | | 0 (0) | | 0 (0) | | 0 (0) |
| Chiroptera | Hipposideridae | 84 | 84 (100) | 0 (0) | 0 (0) | 0 (0) |  | 0 (0) | | 84 (100) | | 0 (0) | | 0 (0) | | 0 (0) |
| Chiroptera | Megadermatidae | 5 | 5 (100) | 0 (0) | 0 (0) | 0 (0) |  | 1 (20) | | 5 (100) | | 0 (0) | | 0 (0) | | 0 (0) |
| Chiroptera | Molossidae | 100 | 100 (100) | 0 (0) | 0 (0) | 0 (0) |  | 0 (0) | | 100 (100) | | 0 (0) | | 0 (0) | | 0 (0) |
| Chiroptera | Mormoopidae | 9 | 9 (100) | 0 (0) | 0 (0) | 0 (0) |  | 0 (0) | | 9 (100) | | 0 (0) | | 0 (0) | | 0 (0) |
| Chiroptera | Mystacinidae | 2 | 0 (0) | 0 (0) | 2 (100) | 0 (0) |  | 0 (0) | | 2 (100) | | 0 (0) | | 1 (50) | | 0 (0) |
| Chiroptera | Myzopodidae | 2 | 2 (100) | 0 (0) | 0 (0) | 0 (0) |  | 0 (0) | | 2 (100) | | 0 (0) | | 0 (0) | | 0 (0) |
| Chiroptera | Natalidae | 11 | 11 (100) | 0 (0) | 0 (0) | 0 (0) |  | 0 (0) | | 10 (91) | | 0 (0) | | 0 (0) | | 0 (0) |
| Chiroptera | Noctilionidae | 2 | 2 (100) | 0 (0) | 0 (0) | 0 (0) |  | 0 (0) | | 2 (100) | | 0 (0) | | 0 (0) | | 0 (0) |
| Chiroptera | Nycteridae | 16 | 16 (100) | 0 (0) | 0 (0) | 0 (0) |  | 0 (0) | | 16 (100) | | 0 (0) | | 0 (0) | | 0 (0) |
| Chiroptera | Phyllostomidae | 174 | 22 (13) | 65 (37) | 87 (50) | 0 (0) |  | 2 (1) | | 69 (40) | | 1 (1) | | 120 (69) | | 0 (0) |
| Chiroptera | Pteropodidae | 187 | 0 (0) | 187 (100) | 0 (0) | 0 (0) |  | 0 (0) | | 0 (0) | | 5 (3) | | 172 (92) | | 2 (1) |
| Chiroptera | Rhinolophidae | 74 | 74 (100) | 0 (0) | 0 (0) | 0 (0) |  | 0 (0) | | 74 (100) | | 0 (0) | | 0 (0) | | 0 (0) |
| Chiroptera | Rhinopomatidae | 4 | 4 (100) | 0 (0) | 0 (0) | 0 (0) |  | 0 (0) | | 4 (100) | | 0 (0) | | 0 (0) | | 0 (0) |
| Chiroptera | Thyropteridae | 4 | 4 (100) | 0 (0) | 0 (0) | 0 (0) |  | 0 (0) | | 4 (100) | | 0 (0) | | 0 (0) | | 0 (0) |
| Chiroptera | Vespertilionidae | 421 | 421 (100) | 0 (0) | 0 (0) | 0 (0) |  | 0 (0) | | 416 (99) | | 0 (0) | | 0 (0) | | 0 (0) |
| Cingulata | Dasypodidae | 21 | 12 (57) | 0 (0) | 9 (43) | 0 (0) |  | 0 (0) | | 19 (90) | | 0 (0) | | 2 (10) | | 2 (10) |
| Dasyuromorphia | Dasyuridae | 72 | 65 (90) | 0 (0) | 7 (10) | 0 (0) |  | 19 (26) | | 61 (85) | | 0 (0) | | 0 (0) | | 0 (0) |
| Dasyuromorphia | Myrmecobiidae | 1 | 1 (100) | 0 (0) | 0 (0) | 0 (0) |  | 0 (0) | | 1 (100) | | 0 (0) | | 0 (0) | | 0 (0) |
| Dasyuromorphia | Thylacinidae | 1 | 1 (100) | 0 (0) | 0 (0) | 0 (0) |  | 1 (100) | | 0 (0) | | 0 (0) | | 0 (0) | | 0 (0) |
| Dermoptera | Cynocephalidae | 2 | 0 (0) | 2 (100) | 0 (0) | 0 (0) |  | 0 (0) | | 0 (0) | | 0 (0) | | 0 (0) | | 2 (100) |
| Didelphimorphia | Didelphidae | 95 | 11 (12) | 2 (2) | 82 (86) | 0 (0) |  | 26 (27) | | 91 (96) | | 19 (20) | | 65 (68) | | 1 (1) |
| Diprotodontia | Acrobatidae | 2 | 0 (0) | 0 (0) | 2 (100) | 0 (0) |  | 0 (0) | | 2 (100) | | 0 (0) | | 1 (50) | | 0 (0) |
| Diprotodontia | Burramyidae | 5 | 0 (0) | 0 (0) | 5 (100) | 0 (0) |  | 0 (0) | | 1 (20) | | 5 (100) | | 5 (100) | | 4 (80) |
| Diprotodontia | Hypsiprymnodontidae | 1 | 0 (0) | 0 (0) | 1 (100) | 0 (0) |  | 0 (0) | | 1 (100) | | 0 (0) | | 0 (0) | | 0 (0) |
| Diprotodontia | Macropodidae | 67 | 0 (0) | 67 (100) | 0 (0) | 0 (0) |  | 0 (0) | | 0 (0) | | 0 (0) | | 21 (31) | | 64 (96) |
| Diprotodontia | Petauridae | 11 | 2 (18) | 1 (9) | 8 (73) | 0 (0) |  | 0 (0) | | 10 (91) | | 0 (0) | | 1 (9) | | 1 (9) |
| Diprotodontia | Phalangeridae | 26 | 0 (0) | 10 (38) | 16 (62) | 0 (0) |  | 0 (0) | | 1 (4) | | 3 (12) | | 22 (85) | | 22 (85) |
| Diprotodontia | Phascolarctidae | 1 | 0 (0) | 1 (100) | 0 (0) | 0 (0) |  | 0 (0) | | 0 (0) | | 0 (0) | | 0 (0) | | 1 (100) |
| Diprotodontia | Potoroidae | 11 | 0 (0) | 5 (45) | 6 (55) | 0 (0) |  | 0 (0) | | 3 (27) | | 3 (27) | | 0 (0) | | 5 (45) |
| Diprotodontia | Pseudocheiridae | 18 | 0 (0) | 18 (100) | 0 (0) | 0 (0) |  | 0 (0) | | 0 (0) | | 0 (0) | | 11 (61) | | 17 (94) |
| Diprotodontia | Tarsipedidae | 1 | 0 (0) | 1 (100) | 0 (0) | 0 (0) |  | 0 (0) | | 0 (0) | | 0 (0) | | 0 (0) | | 0 (0) |
| Diprotodontia | Vombatidae | 3 | 0 (0) | 3 (100) | 0 (0) | 0 (0) |  | 0 (0) | | 0 (0) | | 0 (0) | | 0 (0) | | 3 (100) |
| Eulipotyphla | Erinaceidae | 24 | 13 (54) | 0 (0) | 11 (46) | 0 (0) |  | 3 (12) | | 24 (100) | | 2 (8) | | 5 (21) | | 0 (0) |
| Eulipotyphla | Nesophontidae | 6 | 6 (100) | 0 (0) | 0 (0) | 0 (0) |  | 0 (0) | | 6 (100) | | 0 (0) | | 0 (0) | | 0 (0) |
| Eulipotyphla | Solenodontidae | 3 | 1 (33) | 0 (0) | 2 (67) | 0 (0) |  | 0 (0) | | 3 (100) | | 0 (0) | | 2 (67) | | 0 (0) |
| Eulipotyphla | Soricidae | 376 | 339 (90) | 0 (0) | 37 (10) | 0 (0) |  | 1 (0) | | 373 (99) | | 0 (0) | | 0 (0) | | 0 (0) |
| Eulipotyphla | Talpidae | 41 | 38 (93) | 0 (0) | 3 (7) | 0 (0) |  | 0 (0) | | 41 (100) | | 0 (0) | | 0 (0) | | 0 (0) |
| Hyracoidea | Procaviidae | 5 | 0 (0) | 5 (100) | 0 (0) | 0 (0) |  | 0 (0) | | 0 (0) | | 0 (0) | | 3 (60) | | 5 (100) |
| Lagomorpha | Leporidae | 62 | 0 (0) | 61 (98) | 1 (2) | 0 (0) |  | 0 (0) | | 0 (0) | | 3 (5) | | 3 (5) | | 61 (98) |
| Lagomorpha | Ochotonidae | 30 | 0 (0) | 30 (100) | 0 (0) | 0 (0) |  | 0 (0) | | 0 (0) | | 0 (0) | | 0 (0) | | 16 (53) |
| Lagomorpha | Prolagidae | 1 | 0 (0) | 0 (0) | 0 (0) | 1 (100) |  | 0 (0) | | 0 (0) | | 0 (0) | | 0 (0) | | 0 (0) |
| Macroscelidea | Macroscelididae | 17 | 5 (29) | 0 (0) | 12 (71) | 0 (0) |  | 0 (0) | | 17 (100) | | 0 (0) | | 0 (0) | | 0 (0) |
| Microbiotheria | Microbiotheriidae | 1 | 1 (100) | 0 (0) | 0 (0) | 0 (0) |  | 0 (0) | | 1 (100) | | 0 (0) | | 0 (0) | | 0 (0) |
| Monotremata | Ornithorhynchidae | 1 | 1 (100) | 0 (0) | 0 (0) | 0 (0) |  | 0 (0) | | 1 (100) | | 0 (0) | | 0 (0) | | 0 (0) |
| Monotremata | Tachyglossidae | 4 | 4 (100) | 0 (0) | 0 (0) | 0 (0) |  | 0 (0) | | 4 (100) | | 0 (0) | | 0 (0) | | 0 (0) |
| Notoryctemorphia | Notoryctidae | 2 | 1 (50) | 0 (0) | 1 (50) | 0 (0) |  | 0 (0) | | 2 (100) | | 0 (0) | | 0 (0) | | 0 (0) |
| Paucituberculata | Caenolestidae | 6 | 2 (33) | 0 (0) | 2 (33) | 2 (33) |  | 0 (0) | | 6 (100) | | 0 (0) | | 0 (0) | | 0 (0) |
| Peramelemorphia | Chaeropodidae | 1 | 0 (0) | 0 (0) | 1 (100) | 0 (0) |  | 0 (0) | | 0 (0) | | 0 (0) | | 0 (0) | | 0 (0) |
| Peramelemorphia | Peramelidae | 19 | 3 (16) | 3 (16) | 10 (53) | 3 (16) |  | 0 (0) | | 13 (68) | | 0 (0) | | 7 (37) | | 0 (0) |
| Peramelemorphia | Thylacomyidae | 2 | 1 (50) | 1 (50) | 0 (0) | 0 (0) |  | 0 (0) | | 1 (50) | | 1 (50) | | 0 (0) | | 0 (0) |
| Perissodactyla | Equidae | 7 | 0 (0) | 7 (100) | 0 (0) | 0 (0) |  | 0 (0) | | 0 (0) | | 0 (0) | | 0 (0) | | 7 (100) |
| Perissodactyla | Rhinocerotidae | 5 | 0 (0) | 5 (100) | 0 (0) | 0 (0) |  | 0 (0) | | 0 (0) | | 0 (0) | | 2 (40) | | 5 (100) |
| Perissodactyla | Tapiridae | 4 | 0 (0) | 4 (100) | 0 (0) | 0 (0) |  | 0 (0) | | 0 (0) | | 0 (0) | | 4 (100) | | 3 (75) |
| Pholidota | Manidae | 8 | 8 (100) | 0 (0) | 0 (0) | 0 (0) |  | 0 (0) | | 8 (100) | | 0 (0) | | 0 (0) | | 0 (0) |
| Pilosa | Bradypodidae | 4 | 0 (0) | 4 (100) | 0 (0) | 0 (0) |  | 0 (0) | | 0 (0) | | 0 (0) | | 0 (0) | | 4 (100) |
| Pilosa | Cyclopedidae | 1 | 1 (100) | 0 (0) | 0 (0) | 0 (0) |  | 0 (0) | | 1 (100) | | 0 (0) | | 0 (0) | | 0 (0) |
| Pilosa | Megalonychidae | 2 | 0 (0) | 2 (100) | 0 (0) | 0 (0) |  | 0 (0) | | 0 (0) | | 0 (0) | | 2 (100) | | 1 (50) |
| Pilosa | Myrmecophagidae | 3 | 3 (100) | 0 (0) | 0 (0) | 0 (0) |  | 0 (0) | | 3 (100) | | 0 (0) | | 0 (0) | | 0 (0) |
| Primates | Aotidae | 11 | 0 (0) | 0 (0) | 11 (100) | 0 (0) |  | 0 (0) | | 2 (18) | | 2 (18) | | 11 (100) | | 4 (36) |
| Primates | Atelidae | 28 | 0 (0) | 28 (100) | 0 (0) | 0 (0) |  | 0 (0) | | 0 (0) | | 0 (0) | | 13 (46) | | 16 (57) |
| Primates | Callitrichidae | 42 | 0 (0) | 2 (5) | 40 (95) | 0 (0) |  | 0 (0) | | 37 (88) | | 0 (0) | | 39 (93) | | 0 (0) |
| Primates | Cebidae | 17 | 0 (0) | 0 (0) | 17 (100) | 0 (0) |  | 0 (0) | | 17 (100) | | 6 (35) | | 17 (100) | | 0 (0) |
| Primates | Cercopithecidae | 122 | 3 (2) | 85 (70) | 34 (28) | 0 (0) |  | 0 (0) | | 15 (12) | | 23 (19) | | 75 (61) | | 63 (52) |
| Primates | Cheirogaleidae | 29 | 1 (3) | 0 (0) | 28 (97) | 0 (0) |  | 0 (0) | | 29 (100) | | 0 (0) | | 24 (83) | | 16 (55) |
| Primates | Daubentoniidae | 1 | 0 (0) | 0 (0) | 1 (100) | 0 (0) |  | 0 (0) | | 1 (100) | | 1 (100) | | 0 (0) | | 0 (0) |
| Primates | Galagidae | 18 | 0 (0) | 1 (6) | 17 (94) | 0 (0) |  | 0 (0) | | 14 (78) | | 0 (0) | | 0 (0) | | 0 (0) |
| Primates | Hominidae | 7 | 0 (0) | 4 (57) | 3 (43) | 0 (0) |  | 0 (0) | | 0 (0) | | 0 (0) | | 6 (86) | | 2 (29) |
| Primates | Hylobatidae | 16 | 0 (0) | 9 (56) | 7 (44) | 0 (0) |  | 0 (0) | | 0 (0) | | 0 (0) | | 15 (94) | | 5 (31) |
| Primates | Indriidae | 18 | 0 (0) | 18 (100) | 0 (0) | 0 (0) |  | 0 (0) | | 0 (0) | | 0 (0) | | 10 (56) | | 18 (100) |
| Primates | Lemuridae | 20 | 0 (0) | 20 (100) | 0 (0) | 0 (0) |  | 0 (0) | | 0 (0) | | 0 (0) | | 14 (70) | | 13 (65) |
| Primates | Lepilemuridae | 24 | 0 (0) | 24 (100) | 0 (0) | 0 (0) |  | 0 (0) | | 0 (0) | | 0 (0) | | 0 (0) | | 24 (100) |
| Primates | Lorisidae | 10 | 1 (10) | 1 (10) | 8 (80) | 0 (0) |  | 2 (20) | | 8 (80) | | 0 (0) | | 4 (40) | | 0 (0) |
| Primates | Palaeopropithecidae | 1 | 0 (0) | 1 (100) | 0 (0) | 0 (0) |  | 0 (0) | | 0 (0) | | 0 (0) | | 1 (100) | | 1 (100) |
| Primates | Pitheciidae | 43 | 0 (0) | 11 (26) | 32 (74) | 0 (0) |  | 2 (5) | | 19 (44) | | 26 (60) | | 43 (100) | | 19 (44) |
| Primates | Tarsiidae | 8 | 8 (100) | 0 (0) | 0 (0) | 0 (0) |  | 0 (0) | | 8 (100) | | 0 (0) | | 0 (0) | | 0 (0) |
| Proboscidea | Elephantidae | 2 | 0 (0) | 2 (100) | 0 (0) | 0 (0) |  | 0 (0) | | 0 (0) | | 0 (0) | | 2 (100) | | 2 (100) |
| Rodentia | Abrocomidae | 10 | 0 (0) | 10 (100) | 0 (0) | 0 (0) |  | 0 (0) | | 0 (0) | | 0 (0) | | 0 (0) | | 1 (10) |
| Rodentia | Anomaluridae | 7 | 0 (0) | 3 (43) | 4 (57) | 0 (0) |  | 0 (0) | | 0 (0) | | 0 (0) | | 6 (86) | | 0 (0) |
| Rodentia | Aplodontiidae | 1 | 0 (0) | 1 (100) | 0 (0) | 0 (0) |  | 0 (0) | | 0 (0) | | 0 (0) | | 0 (0) | | 0 (0) |
| Rodentia | Bathyergidae | 15 | 1 (7) | 14 (93) | 0 (0) | 0 (0) |  | 0 (0) | | 1 (7) | | 0 (0) | | 0 (0) | | 9 (60) |
| Rodentia | Calomyscidae | 8 | 0 (0) | 3 (38) | 5 (62) | 0 (0) |  | 0 (0) | | 0 (0) | | 7 (88) | | 0 (0) | | 1 (12) |
| Rodentia | Capromyidae | 19 | 0 (0) | 7 (37) | 12 (63) | 0 (0) |  | 0 (0) | | 0 (0) | | 0 (0) | | 5 (26) | | 8 (42) |
| Rodentia | Castoridae | 2 | 0 (0) | 2 (100) | 0 (0) | 0 (0) |  | 0 (0) | | 0 (0) | | 0 (0) | | 0 (0) | | 2 (100) |
| Rodentia | Caviidae | 18 | 0 (0) | 18 (100) | 0 (0) | 0 (0) |  | 0 (0) | | 0 (0) | | 0 (0) | | 0 (0) | | 11 (61) |
| Rodentia | Chinchillidae | 7 | 0 (0) | 7 (100) | 0 (0) | 0 (0) |  | 0 (0) | | 0 (0) | | 2 (29) | | 0 (0) | | 5 (71) |
| Rodentia | Cricetidae | 698 | 37 (5) | 273 (39) | 388 (56) | 0 (0) |  | 1 (0) | | 391 (56) | | 297 (43) | | 170 (24) | | 274 (39) |
| Rodentia | Ctenodactylidae | 5 | 0 (0) | 5 (100) | 0 (0) | 0 (0) |  | 0 (0) | | 0 (0) | | 5 (100) | | 0 (0) | | 5 (100) |
| Rodentia | Ctenomyidae | 60 | 0 (0) | 60 (100) | 0 (0) | 0 (0) |  | 0 (0) | | 0 (0) | | 0 (0) | | 0 (0) | | 52 (87) |
| Rodentia | Cuniculidae | 2 | 0 (0) | 1 (50) | 1 (50) | 0 (0) |  | 0 (0) | | 0 (0) | | 1 (50) | | 2 (100) | | 1 (50) |
| Rodentia | Dasyproctidae | 13 | 0 (0) | 13 (100) | 0 (0) | 0 (0) |  | 0 (0) | | 0 (0) | | 4 (31) | | 13 (100) | | 7 (54) |
| Rodentia | Diatomyidae | 1 | 0 (0) | 1 (100) | 0 (0) | 0 (0) |  | 0 (0) | | 0 (0) | | 1 (100) | | 0 (0) | | 1 (100) |
| Rodentia | Dinomyidae | 1 | 0 (0) | 1 (100) | 0 (0) | 0 (0) |  | 0 (0) | | 0 (0) | | 0 (0) | | 1 (100) | | 1 (100) |
| Rodentia | Dipodidae | 50 | 2 (4) | 23 (46) | 25 (50) | 0 (0) |  | 0 (0) | | 23 (46) | | 32 (64) | | 12 (24) | | 9 (18) |
| Rodentia | Echimyidae | 89 | 0 (0) | 88 (99) | 1 (1) | 0 (0) |  | 0 (0) | | 0 (0) | | 46 (52) | | 80 (90) | | 38 (43) |
| Rodentia | Erethizontidae | 18 | 0 (0) | 15 (83) | 2 (11) | 1 (6) |  | 0 (0) | | 0 (0) | | 3 (17) | | 13 (72) | | 10 (56) |
| Rodentia | Geomyidae | 39 | 0 (0) | 39 (100) | 0 (0) | 0 (0) |  | 0 (0) | | 0 (0) | | 0 (0) | | 0 (0) | | 13 (33) |
| Rodentia | Gliridae | 28 | 2 (7) | 2 (7) | 24 (86) | 0 (0) |  | 3 (11) | | 24 (86) | | 22 (79) | | 24 (86) | | 0 (0) |
| Rodentia | Heteromyidae | 62 | 0 (0) | 31 (50) | 31 (50) | 0 (0) |  | 0 (0) | | 13 (21) | | 61 (98) | | 10 (16) | | 22 (35) |
| Rodentia | Hystricidae | 11 | 0 (0) | 9 (82) | 2 (18) | 0 (0) |  | 0 (0) | | 0 (0) | | 0 (0) | | 11 (100) | | 0 (0) |
| Rodentia | Muridae | 711 | 57 (8) | 210 (30) | 438 (62) | 6 (1) |  | 0 (0) | | 317 (45) | | 348 (49) | | 280 (39) | | 251 (35) |
| Rodentia | Myocastoridae | 1 | 0 (0) | 1 (100) | 0 (0) | 0 (0) |  | 0 (0) | | 0 (0) | | 0 (0) | | 0 (0) | | 0 (0) |
| Rodentia | Nesomyidae | 60 | 1 (2) | 0 (0) | 59 (98) | 0 (0) |  | 0 (0) | | 60 (100) | | 20 (33) | | 11 (18) | | 1 (2) |
| Rodentia | Octodontidae | 13 | 0 (0) | 13 (100) | 0 (0) | 0 (0) |  | 0 (0) | | 0 (0) | | 4 (31) | | 8 (62) | | 8 (62) |
| Rodentia | Pedetidae | 2 | 0 (0) | 2 (100) | 0 (0) | 0 (0) |  | 0 (0) | | 0 (0) | | 0 (0) | | 0 (0) | | 0 (0) |
| Rodentia | Petromuridae | 1 | 0 (0) | 1 (100) | 0 (0) | 0 (0) |  | 0 (0) | | 0 (0) | | 1 (100) | | 1 (100) | | 1 (100) |
| Rodentia | Platacanthomyidae | 2 | 0 (0) | 2 (100) | 0 (0) | 0 (0) |  | 0 (0) | | 0 (0) | | 2 (100) | | 2 (100) | | 1 (50) |
| Rodentia | Sciuridae | 279 | 1 (0) | 137 (49) | 141 (51) | 0 (0) |  | 0 (0) | | 83 (30) | | 215 (77) | | 184 (66) | | 89 (32) |
| Rodentia | Spalacidae | 21 | 0 (0) | 21 (100) | 0 (0) | 0 (0) |  | 0 (0) | | 0 (0) | | 1 (5) | | 0 (0) | | 9 (43) |
| Rodentia | Thryonomyidae | 2 | 0 (0) | 2 (100) | 0 (0) | 0 (0) |  | 0 (0) | | 0 (0) | | 2 (100) | | 2 (100) | | 2 (100) |
| Scandentia | Ptilocercidae | 1 | 0 (0) | 0 (0) | 1 (100) | 0 (0) |  | 0 (0) | | 1 (100) | | 0 (0) | | 1 (100) | | 0 (0) |
| Scandentia | Tupaiidae | 19 | 2 (11) | 0 (0) | 17 (89) | 0 (0) |  | 1 (5) | | 19 (100) | | 0 (0) | | 1 (5) | | 0 (0) |
| Tubulidentata | Orycteropodidae | 1 | 1 (100) | 0 (0) | 0 (0) | 0 (0) |  | 0 (0) | | 1 (100) | | 0 (0) | | 0 (0) | | 0 (0) |
| **TOTAL** |  | **5364** | **1637 (31)** | **1926 (36)** | **1788 (33)** | **13 (0)** |  | **209 (4)** | | **2821 (53)** | | **1204 (22)** | | **1692 (32)** | | **1473 (27)** |
|  |  |  |  |  |  |  |  |  | |  | |  | |  | |  |

**References**
